# An IoT-based real-time smart metering deployment for grid optimization: A case study of GEPCO, Pakistan

**DOI:** 10.1371/journal.pone.0338389

**Published:** 2025-12-18

**Authors:** M. Usman Saleem, M. Hamza Tahir Bajwa, Saif Ur Rahman, Huiqing Wen, M. Arif Khan

**Affiliations:** 1 Department of Computer Science, Govt. College Women University, Sialkot, Pakistan; 2 Gujranwala, Electric Power Company, Gujranwala, Pakistan; 3 School of Advanced Technology, Xi’an Jaiotong Liverpool University, China; 4 School of Computing, Mathematics and Engineering, Charles Sturt University, Australia; Aalto University, FINLAND

## Abstract

The increasing demand for energy and the growing environmental issues in Pakistan, require a movement to a more environmentally friendly and smarter energy infrastructure. This work provides the practical application of research which represents the deploying of a smart metering network in real time (RT) in Pakistan’s transition to more environmentally friendly and smarter energy systems. The work presents the design, implementation and the results of the operation of the smart metering deployment implemented by Gujranwala Electric Power Company (GEPCO). The presented system developed on a four-layer Internet of Things (IoT)-based architecture comprising of Energy Monitoring, Communication, Cloud Analytics, and Application layers. The smart meters (SMs) on the three classes of industrial loads (< 50 kW, 50–500 kW, > 500 kW) transmit RT data of the electrical parameters, including voltage, current, power factor, frequency, and consumption, to a centralized meter data management system (MDMS). This data enables the MDMS to support various functions such as automated billing, load profiling, fault detection, and power quality (PQ) analysis. Results of the case studies demonstrate that RT monitoring can assist in attaining a higher degree of grid visibility and operational responsiveness. One such case was the identification of the low power factor (PF) situations (below 0.7) which enabled the deployment of capacitors banks, resulting in measurable energy saving and cost saving in accordance with the mitigated exposure to PF penalties. For instant, in a one large-industrial scenario, PF improved from 67.5% ± 11.2 to 93.6% ± 2.4, corresponding to a significant Welch’s t large effect size, and with reduced day-to-day variability. Moreover, early detection of voltage imbalance, variance of frequencies, and daily peak load patterns were detected using the system. Using a conservative normalize-then-scale approach, a potential average PF uplift of approximately 1.4 percentage points across the industrial segment is projected under stated coverage and adoption assumptions. The results confirm that IoT-enabled smart metering can serve as a practical tool for demand side management (DSM), loss reduction and grid optimization. Finally, the study outlines key technical enablers, policy considerations, and institutional requirements for large-scale smart grid (SG) implementation and offers a replicable framework for developing economies pursuing energy system modernization.

## 1. Introduction

The present power network of Pakistan is inefficient, aging, and prone to numerous faults, rendering it incapable of meeting the growing energy demand [1,2]. Pakistan’s power system entirely focuses on increasing generation to meet the growing demand, and utilizes resources of conventional energy to generate power, such as thermal energy derived from fossil fuels [3]. As fossil fuels are irreversible in nature, such as coal, crude oil, and natural gas, numerous problems arise, including environmental concerns such as emissions of carbon dioxide, the effect of greenhouse gases (GHGs), global warming, erratic weather system patterns [4,5]. economic loss, and rising tariff rates [6,7]. Generally, thermal power plants that rely on fossil fuels have costly power generation [8,9] and the costly large-scale generations ultimately lead to an unsustainable power sector.

Many developed and developing nations have made efforts to attain sustainable development in their power sectors in the past few years. Although some countries face numerous obstacles in achieving their sustainability objectives owing to technical, non-technical, or managerial obstacles, the management of the constantly rising demand for electricity appears to be one of the most pressing issues for both developed and developing nations [10]. The rise in demand for electricity prompted a supply-demand gap and several related problems. Consequently, the suppliers of electricity invested in extra infrastructure and tried to fulfill peak demand by enhancing the grid’s capacity by installing more power plants and transmission systems. While this strategy substantially lowered the supply-demand gap, this approach proved to be an ineffective and expensive operating model as it negatively impacted the general viability of the power sector, and led to enormous financial strain, particularly for developing nations like Pakistan [11,12].

The ineffectiveness of the conventional approach prompted the creation of a novel approach that relies on the notions of energy conservation and management. The primary objective of this approach was to minimize peak demand. Since peak demand on the electricity grid occurs for just a few hours of the day, it would be simpler and more cost-effective if the peak demand throughout the day could be altered, decreased, or switched by managing the loads [13]. The access to day-to-day energy consumption patterns is prerequisite of implementing this novel approach since peak demand can only be managed once consumption patterns are readily available for energy awareness and analysis. Smart meters (SMs) are, therefore, key to this novel approach, that do not only monitor energy parameters but also enable its remote access.

SMs are electronic devices that monitor consumption at frequent intervals and interact with the energy provider. It transmits billing and monitoring data to and acquires control data from the vendor, respectively. Such meters have been installed for the remote recovery of consumption information for billing purposes, as well as an attempt to manage demand. Recently, several international institutions have urged the Pakistani government to implement smart metering technology as soon as possible in order to end the current economic downturn and attain sustainability [14]. Meanwhile, the persistent electricity crisis has posed a substantial obstacle to the achievement of sustainability goals. With the goal of evaluating the viability of the recommendation, this study examines how SMs can help the energy sector of Pakistan in attaining sustainability, challenges that may involve and the way forward. Based on the investigation and worldwide technological patterns in the power sector, it has been established that a smart metering system will be a viable means of addressing Pakistan’s electricity crisis and achieving a sustainable power sector [15].

This study evaluates an Internet of Things (IoT)–based smart metering deployment at Gujranwala Electric Power Company (GEPCO) and examines how real-time (RT) meter data supports distribution-level grid optimization.

We address the following research questions:

1. Deployment & Integration: How can an IoT-enabled smart metering infrastructure be deployed and integrated with GEPCO’s existing utility systems, including the Meter Data Management System (MDMS), to enable reliable RT data acquisition?
2. Operational Insights: What are the operational insights that can be extracted from SM data for monitoring power quality (voltage/frequency), power factor (PF) and load behavior for industrial consumers?
3. Quantified Benefits: To what extent do these insights translate to quantifiable improvements (i.e., voltage compliance, PF improvement and peak period load share) to inform grid operations decisions.
4. Practical Constraints: What are the constraints for the utility scale deployment and how can they be overcome in the context of GEPCO?

These research questions assisted with the design and analysis in this study. The aims and contributions of this work are summarized as below.

Comprehensive Assessment of Pakistan’s Power Sector: This work gives a detailed overview of the current power infrastructure in Pakistan and identifies inefficiencies and sustainability issues in the sector.

Technical Overview of Smart Metering Technology: This dives into how we moved from electromechanical meters to modern SMs, provides their architecture, and their ability to grab RT data while giving a full view of the whole system.

Field Implementation and Case Study at GEPCO: The paper presents an applied case study of smart metering deployment within the GEPCO. The implementation features a four-layer IoT-based network integrated with a MDMS to ensure secure communication, validation, and centralized data handling.

Power Quality and Load Behavior Analysis: Drawing from RT data on three categories of the industrial loads, this study highlights how SMs stand out in tracking PF, voltage compliances, and load patterns, while also enhancing billing precision and fault detection.

Quantified Benefits for Grid Efficiency and Sustainability: The study lays out how SMs make a big difference, giving clearer insight into the grid, lowering technical losses, and improving operational efficiency. They’re also a game-changer for demand-side management (DSM) programs and help optimize resources across the network, with strong potential in places like Pakistan.

Identification of the Challenges and Roadmaps: The study digs into the technical, economic, and regulatory hurdles of rolling out SMs, offering practical solutions like boosting public awareness, building capacity, shaping smarter policies, investing in infrastructure, and engaging consumers to pave the way for success.

Eco-Friendly Upsides and Sustainability Angle: The study spotlights how smart metering technology delivers real environmental wins, like lowering greenhouse gas emissions and reducing dependence on fossil fuels, by boosting energy savings and leveraging data to optimize operations for more intelligent electricity consumption.

The remainder of this paper is organized as follows: Section 2 gives an overview of Pakistan power sector, i.e., current status of power generation, transmission and distribution infrastructure and challenges faced by the power sector. Section 3 deals with smart metering technology by providing a detailed look at the evolution of meters from the conventional meters to the advanced SMs and explains in detail their functionalities and importance for modern power system. Additionally, section 3 briefly provides the advanced metering infrastructure (AMI) deployments in developing grids, MDMS data quality practices, distribution side analytics from SM data and AMI security/privacy considerations. Section 4 is a detailed case study of SMs implementation in the GEPCO. It includes network architecture, MDMS, analytics capability and power quality monitoring. Several industrial consumer case studies show how SM improves load management, and PF improvement. Section 5 describes the advantages associated with SMs in the electricity sector of Pakistan such as load profiling, grid optimization, energy shortage mitigation and environmental benefits. Section 6 discusses challenges in implementation of SMs in Pakistan and suggest of the future directions to ensure successful deployment. Section 7 concludes the paper by summing-up the key findings and the importance of smart metering in the transition of the power sector in Pakistan towards sustainability.

## 2. Overview of the Pakistan’s power sector

In Pakistan, the power sector operates under the Ministry of Water and Power (MoW&P), with the National Electric Power Regulatory Authority (NEPRA) serving as the regulatory body. Electricity generation relies on a diverse mix of energy sources, including thermal (such as natural gas, coal, residual fuel oil, and furnace oil), hydroelectric, nuclear, and renewable sources like solar, wind, and bagasse.

**Fig 1:**
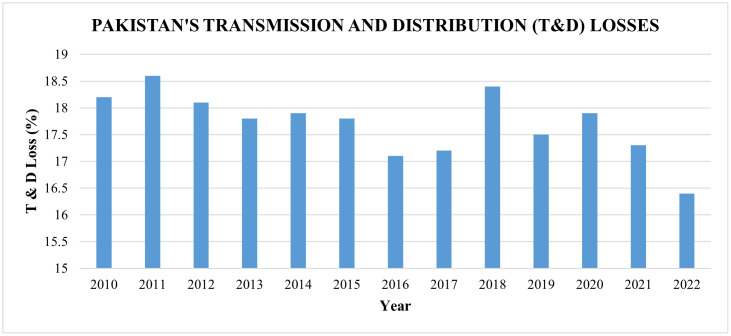
Pakistan’s transmission and distribution losses since 2010 [16].

The transmission of electricity is managed by two key entities, i.e., the National Transmission and Dispatch Company (NTDC) and K-Electric, both playing critical roles in ensuring the reliable delivery of power across the country. NTDC implemented a transmission system of 8097 km with a 500 kV operating voltage and an infrastructure of 11519 km with a 220 kV operating voltage, with eighteen 500 kV substations and forty-nine 220 kV substations [16]. Within the next decade, NTDC plans to add 36,292 MW to the national power grid [17]. Pakistan suffers from significant transmission losses due to its outdated transmission system and lack of funding for energy infrastructure as a result of the country’s unstable political situation. The NTDC aims to minimize loss during transmission by developing cutting-edge technologies that allow them to offer distribution companies reliable and secure power transmission. It intends to construct a 765 KV AC transmission network with a length of 254.5 kilometers for the extraction of electricity from the Diamer Bhasha, Dasu, and Mohmmand hydropower projects [18], and has also inaugurated a 660 kV HVDC transmission network of 878 kilometers from Matiari to Lahore with converter stations at both ends [19,20] with aim to improve its transmission and decrease the losses. On the other hand, eleven distribution companies (DISCOs) in Pakistan handle the distribution of electricity. There are 10 public sector DISCOs and one private vertically incorporated entity, K-Electric. There seems to be a trend of inconsistency in DISCO distribution losses. However, 2022 had the lowest distribution losses, at 16.9%, due to the rigorous monitoring and distribution loss targets set by NEPRA for DISCOs. The transmission and distribution (T&D) losses since 2010 are depicted in Figure 1 [16].

**Fig 2:**
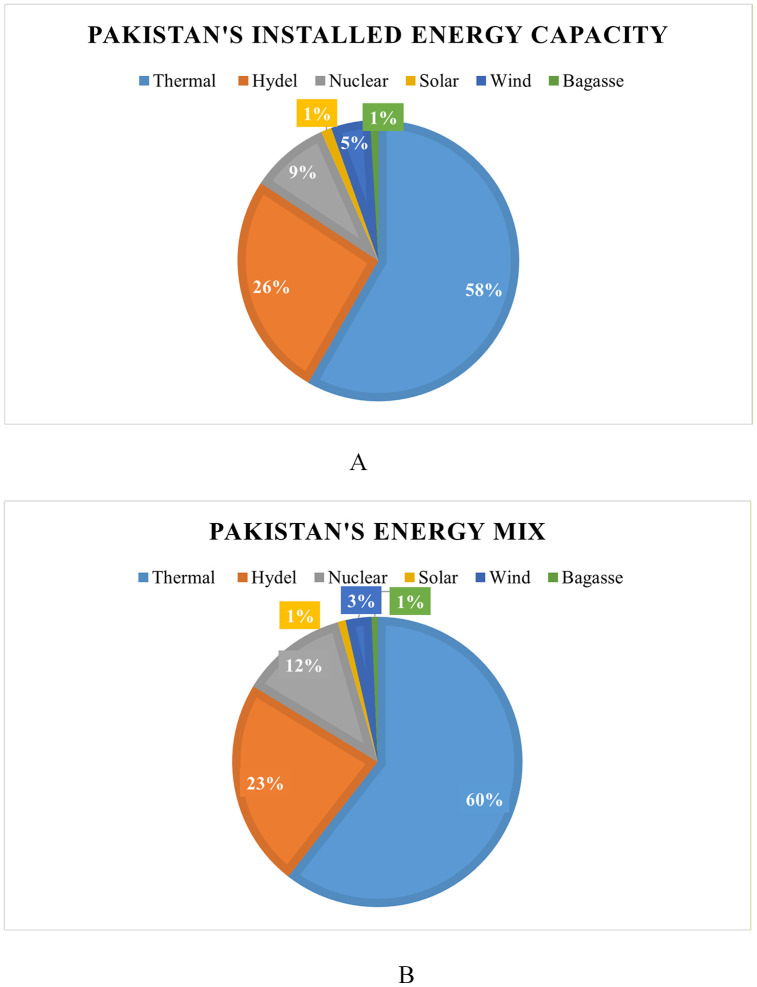
Pakistan’s energy profile. **(A)** Installed energy capacity, **(B)** Energy generation [16].

Currently, there are 4 public Generation Companies (GENCOs) [21,22] and 110 Independent Power Producers (IPPs) [23] with an overall installed capacity of 43,775 MW, which includes an allocation of 25039 MW for IPPs and GENCOs as shown in Table 1. During 2021−22, this newly installed capacity produced approximately 153,874.20 GWh [24]. In 2021−22, thermal power plants (PPs) accounted for 58.29% of the installed capacity and about 60.79% of the overall produced energy. However, hydroelectric power generation accounted for 23.29% of the overall energy generation and 26.00% of the installed capacity. Other sources of power, such as solar, bagasse, wind, and nuclear, contributed 0.47%, 0.65%, 2.87%, and 11.93% of energy generation and 1.23%, 0.89%, 4.52%, and 9.05% of installed capacity, respectively, as shown in Fig 2A and Fig 2B [16].

**Table 1:** Installed capacities (MW) of GENCOs and IPPs.

GENCOs	IPPs hydel	IPPs thermal	IPPs (KE owned)	Total
4,731	1,192	18750	366	25,039

The power grid in Pakistan struggles with significant hurdles in operations, capacity planning and overall management [25]. Through a thoughtful strategy that integrates information and communications technology (ICT) with renewable energy sources (RESs), the existing system could evolve into a smarter grid. Such ICT integration would notably enhance visibility and control over power flows [26]. The SMs serve as a foundational element of ICT and embracing them could represent a pivotal advance in pursuing sustainable development.

## 3. Smart metering technology and evolution

The meter reading is essential for the distribution of the power network and the retailing of power. Analog meter reading was highly laborious, time-consuming, expensive, and inconsistent. Previously, consumers were billed based on an estimate, and the remaining balance was sent after the reading was taken. In addition, the identification of the low-voltage issue was slow as it depended on the consumer’s phone complaint. However, the technology of meter reading has evolved over time. Occasionally, extra features are introduced, such as shifts from analogue metering technology to automatic meter reading to smart metering. The next subsection examines the development of SMs.

### 3.1. Evolution of metering technologies

**Fig 3:**
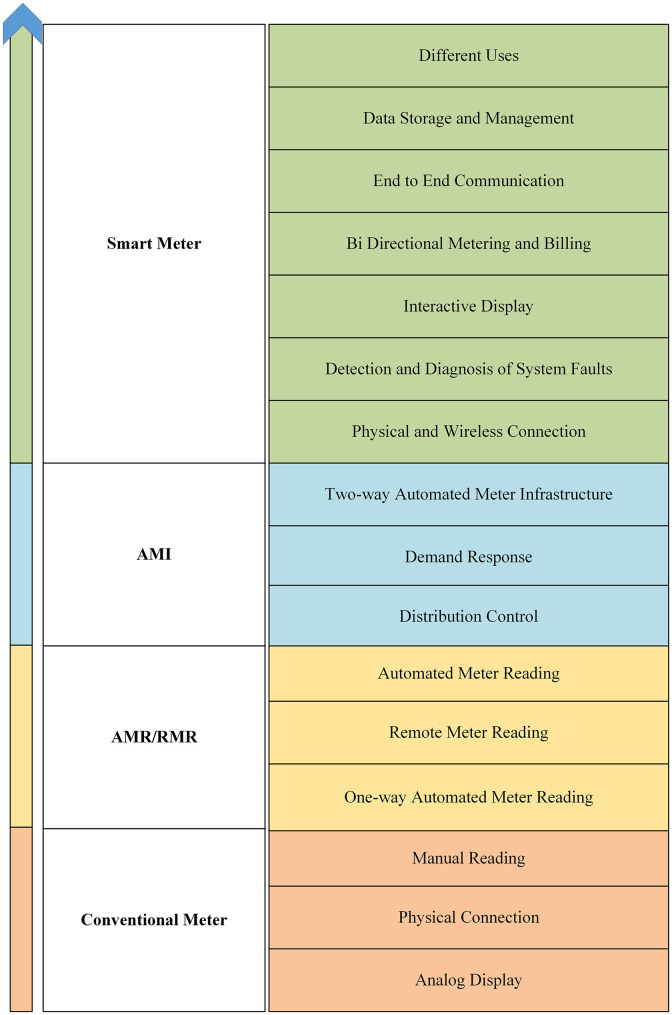
Evolution of the SMs [11].

This section offers the evolution of conventional meters to SMs as depicted in Fig 3 [11].

#### 3.1.1. Electro-mechanical meters.

The electronic mechanical watt-hour meter is the first and most widespread type of electrical meter. Two coils that are inductive in nature and a spinning metallic disc are included. As electrical current flows through the inductive coils, magnetic flux is generated, causing the disc to rotate. The velocity of the disc’s rotation is dependent on the power, and in order to bill customers for their power consumption, the number of disc revolts is measured. The popularity of electromechanical meters was due to their simplistic design, despite their lack of additional characteristics. The invention of electronic meters was necessitated by the need for a more effective control and monitoring system for electricity meters in power grids, necessitated by technological progress.

#### 3.1.2. Electronic-digital meters.

Digital micro-technology is utilized in the construction of electronic meters, obviating the need for electromechanical meters’ heavy movable parts. Due to their digital nature, these meters are more precise and lay the groundwork for SMs. As technology advanced, additional features were added to render them smarter, providing benefits for customers as well as vendors. In addition, providers were capable of supplying electricity effectively by installing load leveling and RT pricing via remote monitoring operations. The development of electronic digital meters enabled the addition of novel functions by simply inserting additional modules.

Remote meter reading (RMR) and automated meter reading (AMR) were made possible by the 1st generation of electro-digital meters, which could record power consumption for the vendor. Therefore, energy providers would be able to access data regarding energy consumption with no human interference from great distances. This prompted the development of a second generation of digital electronic meters with modern metering infrastructure that could handle energy requirements.

The AMI includes the transfer and retention of data via communication between servers and data connections for the purpose of monitoring intelligent energy consumption. This resulted in the creation of SMs, thereby making the proposal of SG possible through the addition of smart features such as instrument control and tracking, two-way communication and billing purposes, storage of data and management of loads, detection of system defects and energy thefts, smart city development, safety and protection enhancement, and emission reduction.

#### 3.1.3. Smart meters.

SMs track power consumption and frequently send data to the centralized information server for control, monitoring, and assessment, leading to the establishment of a reliable and effective smart energy management system (SEMS). Unlike conventional meters, SMs are able to measure various grid parameters, including current, voltage, active and reactive power, frequency and PF. The data recording interval for these grid parameters may range from a few minutes to hours. The record offers an overview of the load usage behavior. Load forecasts frequently result in ineffective DSM, but because of the SMs, efficient, reliable, and successful DSM approaches can be planned. In addition, SMs trigger notifications and alarms whenever an electrical variable deviates from its normal limit. These alerts aid in detecting and eliminating system deficits and electricity losses, thereby facilitating effective load management, fault evaluation, and load profiling.

**Fig 4:**
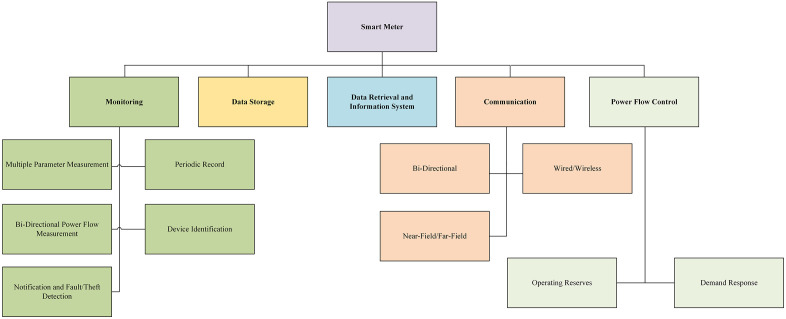
Functionalities of typical SM [11].

Periodically, the data from the SM is transmitted to the centralized server, to make it accessible. Based on the utility’s distance from the central server, the communication system enables the SMs and the server itself to transmit data via a wired or wireless system. There is probably an array of primary and secondary hyperlinks for data transfer in a network of communications that offers accuracy and resilience. Generally, bidirectional data transfer in SM allows RT monitoring and control of diverse electrical loads. The Functionalities of typical SM is shown in Fig 4.

### 3.2. AMI deployments in developing grids

Large emerging markets have transitioned from pilot projects to national SM rollouts, exposing both technical and organizational constraints such as procurement, consumer engagement, interoperability, and data-use capacity. India’s RDSS/AMISP program provides a large-scale reference point; the ISGF white paper synthesizes lessons from multiple DISCOMs and vendors [27,28]. As a mature benchmark, the European Commission JRC monitors EU-wide deployment as a policy and technology reference for interoperability and governance [29,30]. The IEA further summarizes SG progress across emerging markets and developing economies, highlighting AMI’s role in loss reduction and operational visibility [31]. Despite accelerating deployments, few utility-side studies in developing regions report granular operational metrics such as voltage-compliance, PF behavior and peak-window energy shares. This study helps close that gap through an operational GEPCO deployment with quantified industrial-site data.

### 3.3. MDMS and VEE workflows as enablers of analytics

Data quality underpins all operational analytics. In utilities, Validation–Estimation–Editing (VEE) routines within the MDMS verify timestamp alignment, value ranges, spikes, and missing intervals. Canonical VEE rule classes and parameters are documented by Oracle Utilities Meter Data Management [32]. Similar approaches have been emphasized in recent utility-operations frameworks using AMI data for RT monitoring and fault detection [33].

Section 4.1.1 details GEPCO’s VEE configuration, time-alignment checks, range verification, PF thresholds, and exception flags and outlines.

### 3.4. Smart-meter data analytics for distribution operations

Recent research demonstrates how AMI data supports distribution-side decision-making: short-term load forecasting, non-technical-loss detection, topology and impedance inference, asset-health monitoring, and operational-compliance tracking. A review in Renewable and Sustainable Energy Reviews presents a comprehensive taxonomy of such applications [34], while a recent work surveys data-driven analytics for fault detection, forecasting, and anomaly detection [8]. Field evidence from developing utilities, such as an IoT-based SM DSM study in Bangladesh [35], shows feasibility under constrained conditions. Broader analyses highlight the policy relevance of high-frequency AMI data for energy-economics modeling [36].

This paper focuses on the operational slice of that taxonomy, voltage-compliance, PF behavior, and peak-window load shares by using live GEPCO industrial data to demonstrate measurable operational insights.

### 3.5. Security, privacy, and governance of AMI data

As AMI scales, cybersecurity and privacy become central. Vulnerabilities include eavesdropping, denial-of-service, meter tampering, and privacy leakage from fine-grained load traces. A recent survey reviews catalogs these threats and countermeasures, emphasizing encryption, key-management, and anonymization [37,38]. Section 6 briefly discusses governance and future directions for secure AMI deployment.

## 4. Smart metering implementation in Pakistan: A case study of GEPCO

**Fig 5:**
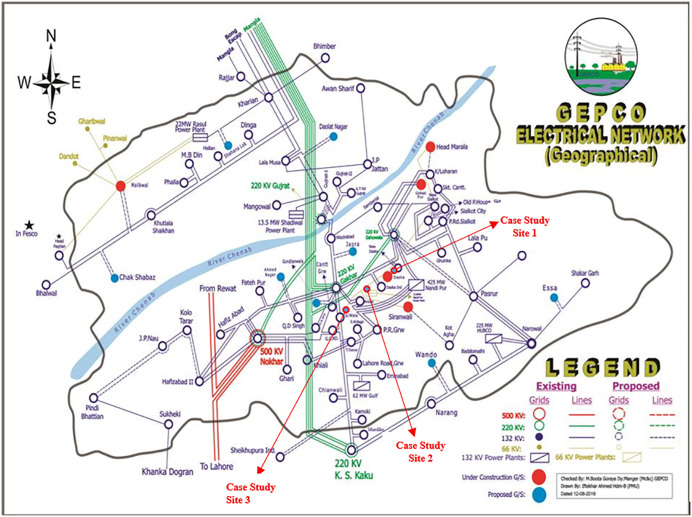
Electrical network of GEPCO [39,40]. Location of case studies indicated.

For case study purposes, the application of SMs in GEPCO is discussed here in this study, and we have analyzed the possible improvements in the GEPCO system that may be achieved through its implementation. GEPCO’s electrical network is comprised of 66 KV and 132 KV grid stations (GSs), which are administered by Manager GSO. There are sixty-one 132 KV GSs with a total length of 2554 km of transmission lines and one 66 KV grid station with a transmission line of 162 kilometers. GEPCO’s distribution infrastructure consists of 949 feeders, with 11 KV lines extending 24,996 kilometers and LT lines measuring 18,496 kilometers. There are nearly 181 power transformers and 80,085 distribution transformers of different capacities. The entire GEPCO’s electrical network is depicted in Fig 5 [40].

**Fig 6:**
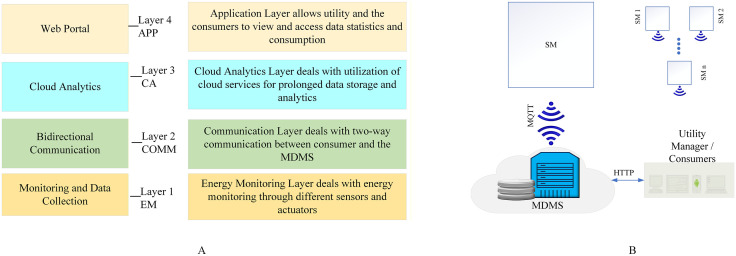
System architecture of the GEPCO platform. **(A)** Overall system architecture of GEPCO; **(B)** Four-layered architecture of GEPCO.

GEPCO has started the installation of SMs for its industrial consumers. The SMs gather the data then transmit to the MDMS, where it is then possible to access the metering data at any time through web servers or consumer application. Fig 6A presents the overall system architecture, while Fig 6B depicts the four-layered architecture of GEPCO. The bottom-most layer is the energy monitoring (EM) layer, which, as the name suggests, deals with EM and measures and monitors various energy parameters through numerous actuators and sensors. The second layer from the bottom is the communication (COMM) layer, which plays its role in two-way communication between MDMS and consumers and utilizes the IoT protocol for this data transfer. The third layer is the cloud analytics (CA) layer, which deals with the utilization of cloud services and facilitates data processing, analytics, and storage. The fourth one is the application (APP) layer, which involves the interaction between the cloud server and the consumers. It enables utilities or consumers to access stored data to view energy consumption or other statistics.

### 4.1. Overview of GEPCO’s Meter Data Management System (MDMS)

GEPCO utilizes KBK’s MDMS solution, which is a robust platform that maximizes the worth of meter data and enables utilities to forecast, analyze, and execute flawlessly. It collects and processes data from SM using numerous methods, such as Multi-Speak, Common Information Model, Table Access, Manual File Import, Web Services, and File Transfer Protocol (FTP). It incorporates, processes, stores, validates, and displays data in a variety of ways to facilitate operations through application later.

**Fig 7:**
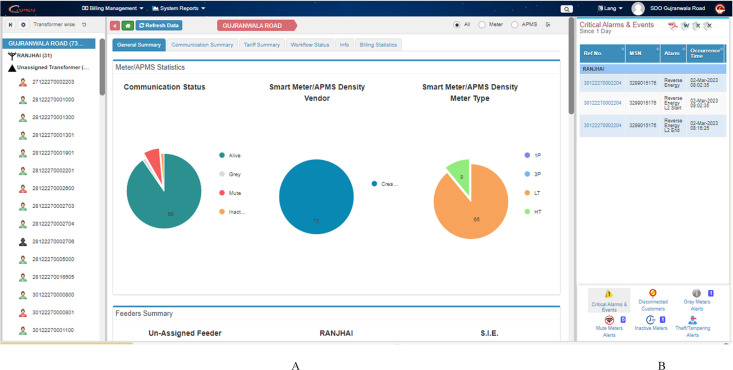
User interface for data access and alert visualization in the PITC project. **(A)** PITC project window for accessing MDMS data; **(B)** Alert panel showing SM alerts.

The data in MDMS can be accessed through the Power Information Technology Company (PITC) website, whose project window is given in Fig 7A It enables getting billing reports at any time and makes it possible to monitor and watch the consumption of any of the consumers at any instant. The left side of this window shows a complete list of connections where SMs have been installed, while its right side shows 6 different types of alerts, as shown in Fig 7B. In case any suspiciousness is observed or reverse energy flows through the energy meter, the utility gets alerts in the ‘Critical Alarms and Events’ pane. The disconnected energy connections are shown in the ‘Disconnected Costumers’ pane, while the customers who have active energy connections but whose consumption remains zero are shown in the ‘Inactive Meters’ pane. The SMs whose communication with the server is interrupted and stops are first kept by MDMS in the ‘Gray Meters’ list, where MDMS waits for 48 hours and checks if this interruption in communication is temporary, which may be due to network problems. However, if their communication does not resume within 48 hours, the meters are sent to the ‘Mute Meters’ pane, which alerts the utility, and, responding to communication failure, the utility performs physical site checking to check and resolve the problem. The last and most important alert is the ‘Theft and Tampering’ alert which notifies the utility if a consumer attempts to tamper with the meter and hence facilitates better theft control, without which energy sustainability can never be attained.

#### 4.1.1 Data validation, estimation, and editing (VEE) process.

The SMs deployed at GEPCO generate time-stamped measurements of all grid parameters that are transferred to the MDMS. To keep the data ready for analysis, a VEE process was used to fix issues like missing data and communication gaps [32].

1 Timestamp and sequence verification

Each record of 15-minute interval was checked for duplication and sequencing. Out-of-order or repeated timestamps were flagged, and only the most recent valid record was retained.

2 Range and logical checks

Readings were examined for acceptable operating ranges consistent with GEPCO’s network parameters:

- Voltage between 0 V and 300 V for low-voltage feeders and 6 kV – 7 kV for 11 kV feeders.
- Power factor values constrained to the physical range 0 ≤ PF ≤ 1.
- Consistency among voltage, current, and power values verified through expected three-phase relationships.

Records outside these limits were automatically flagged for review.

1. Handling of missing or incomplete data
  - Short gaps (one or two missing intervals) were interpolated linearly between adjacent valid points.
  - Moderate outages (up to one day) were estimated using the most recent day with similar load characteristics (weekday/weekend pattern).
  - Extended outages (greater than one day) were left unfilled but retained as missing, clearly marked as “communication outage” periods.
2. Outlier detection and adjustment

Sudden spikes or drops inconsistent with normal operating behavior were detected using threshold-based deviation rules. Out-of-range points were replaced by the nearest valid boundary value and flagged as edited.

1. Quality flags and auditability

Each record was assigned a status flag; valid, estimated, edited, or missing, allowing complete reconstruction of the validation history. Periodic manual checks were conducted for meters showing frequent communication issues to confirm data accuracy.

### 4.2 Operational flow and components of the smart meter

**Fig 8:**
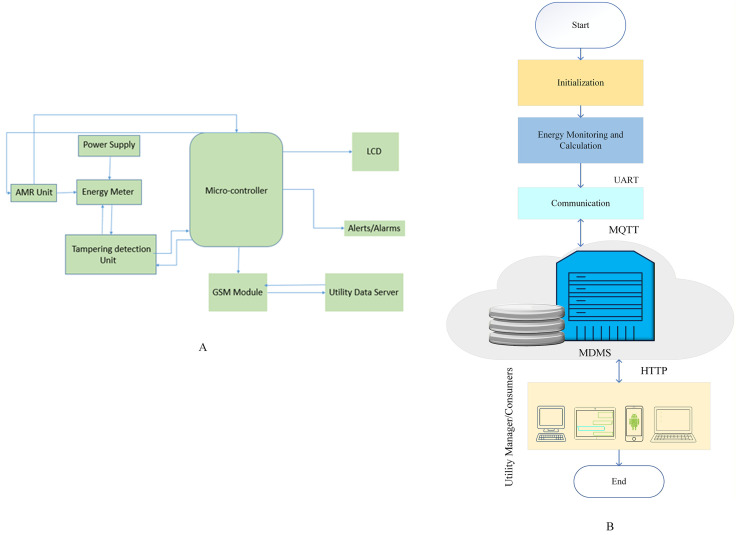
Hardware architecture and operational workflow of the Smart Meter. **(A)** Block diagram of the Smart Meter (SM); **(B)** Operational flow chart of the Smart Meter.

The main components of the SM are the energy meter, microcontroller, AMR unit, tampering detection unit, GSM/GPRS module, and LCD. The AMR unit contains CTs and voltage sensors to measure current and voltage. The microcontroller is an essential component of a SM, which is linked with all other main modules. It assists in recording meter readings, storing the data that was measured and alerting about theft. The measured energy variables are then sent to the MDMS using the GSM/GPRS module and are also displayed on the LCD of the energy meter. This GSM/GPRS module has a slot whereby a SIM card can be inserted and a radio antenna, which links it to the GSM network. This module connects to the internet and communicates with the GSM network using cellular infrastructure. These SMs also contain a meter-tampering detection unit that consists of electromagnetic sensors, a strong anti-magnetic mechanism, and vibration sensors. It enables this unit to detect and block any magnetic force that tries to hinder the working of the meter. Additionally, if anyone tries to open and tamper with this meter, this unit will detect the vibrations and generate a signal or alarm. Thus, if a consumer tries to tamper with the meter, the meter-tampering detection unit will detect and identify any such activity and send an alert to the utility. The meter also sends other alerts if its communication with the utility is interrupted for any reason. The block diagram of SM is illustrated in Fig 8A, whereas the operational flow chart is shown in Fig 8B.

### 4.3 Enhancing sustainability with MDMS-based analytics

The MDMS solution can be utilized to perform various analytics such as billing analytics, load profiling, demand response evaluation, outage management, PQ analytics, revenue protection and customer behavior analytics. Out of these analytics, billing analytics, load profiling, PQ analytics, and consumer behavior analytics have been performed in this study to achieve research objectives of achieving a sustainable electricity sector. Billing analytics ensures accurate billing based on actual energy consumption and addresses overbilling issues. These accurate bills promote transparency and provide accurate data on energy consumption patterns. On the other hand, load profile analytics help identify consumption patterns and peak load times, which helps grid optimization and decrease load on the grid, thereby leading to increased efficiency and attaining sustainability in the electricity sector.

PQ analytics identify and analyze the issues in the distribution system that cause energy losses, such as excessive reactive power flow, unbalanced loads, low PF, voltage drops, etc. By analyzing and monitoring PQ parameters we can improve energy efficiency and cut overall demand. Furthermore, consumer behavior analytics also contribute to achieving sustainability by identifying trends in energy consumption and power saving opportunities. These analytics help create energy awareness and promote energy conservation.

### 4.4. Case studies of smart meter deployment in industrial consumers

In this section, power analytics of various industrial consumers under the jurisdiction of GEPCO have been performed. For the said purpose, we have divided the GEPCO’s industrial consumers into three different categories based on their sanctioned loads. The first category includes industries that have sanctioned loads less than 50 kW, while the second and third categories include industries with loads less than 500 kW and industries with sanctioned loads greater than 500 kW. Afterwards, three of the representative consumers, one from each category, have been selected and analyzed in this paper. The criterion for the selection is as follows:

1. Representation of load categories: Consumers were chosen from different sanctioned-load groups so that each major class of industrial demand within GEPCO’s network was represented.
2. Data completeness and reliability: Only consumers with continuous SM data for a period of at least six months and with communication uptime exceeding 95% in the MDMS (after VEE checks) were included.
3. Geographical accessibility: Preference was given to consumers located within or near the researchers’ locality, allowing timely field verification and meter inspection when required.

#### 4.4.1. Case study I: Load category < 50 kW..

**Fig 9:**
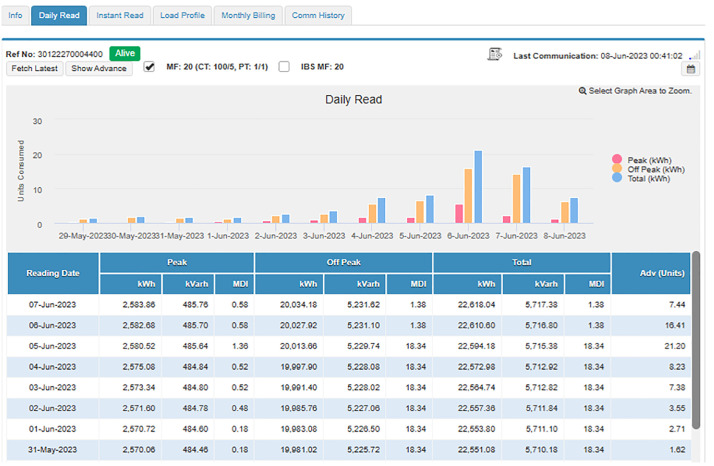
Daily readings of peak, off-peak and total units.

**Fig 10:**
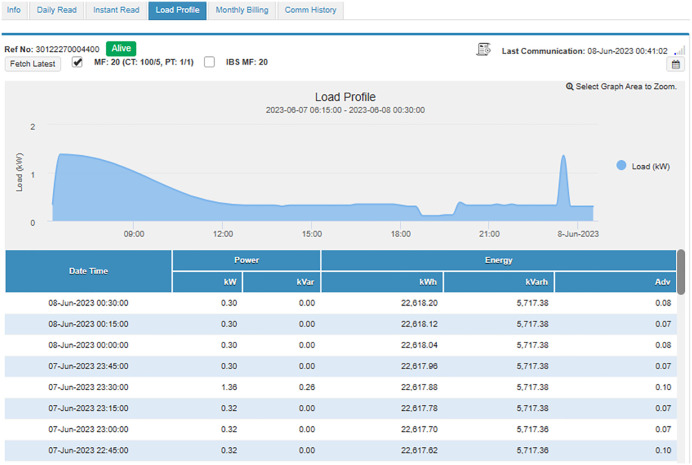
Load profile of SM connection having load < 50 kW.

**Fig 11:**
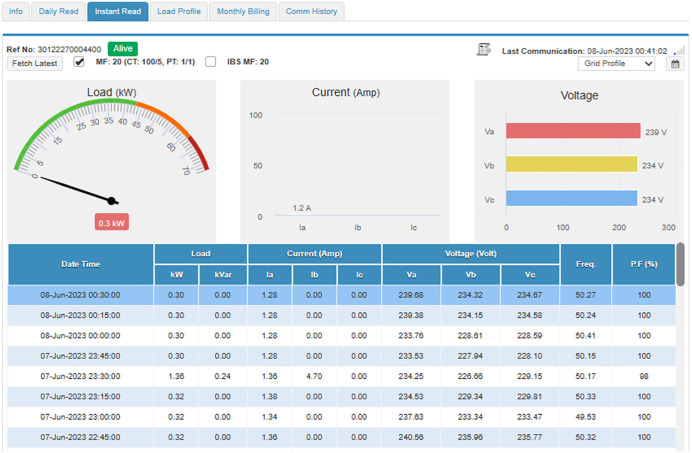
Instantaneous readings of PQ parameters for load< 50 kW.

**Fig 12:**
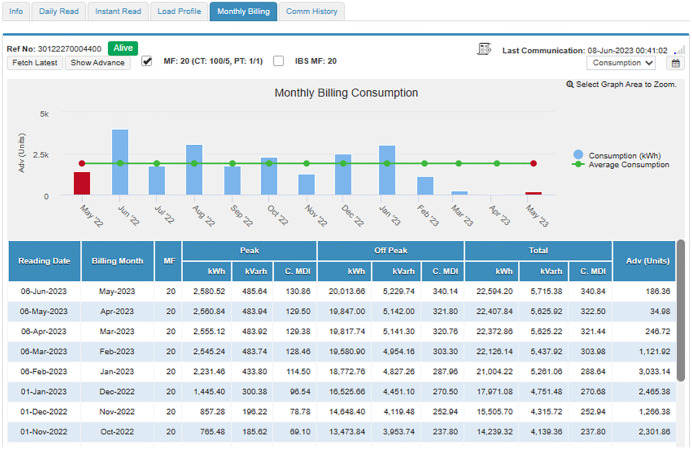
Monthly billing through for load< 50 kW.

In this subsection, an industrial connection with a sanctioned load below 50 kW is studied and analyzed. The connection bearing Reference Number 30 12227 0004400 registered in the name of Dr. Masood Ahmed Cheema S/O Dr. Sultan Ahmed Cheema has been selected for this purpose, has a sanctioned load of 37 kW, CTs of 100/5, and is running in Tehsil Daska, District Sialkot. Its daily readings of peak and off-peak units, and load profiles are available and can be accessed through MDMS, as shown in Fig 9 and Fig 10, respectively. In addition, as shown in Fig 11, the instantaneous readings of its load, voltages, current, PF, and frequency are periodically stored in MDMS at a 15-minute interval. The availability of this periodic data makes the consumer energy aware, discourages energy waste, and encourages energy conservation. Moreover, its monthly billing, shown in Fig 12, also takes place through MDMS, facilitated by the SM. These billing analytics ensure timely and accurate monthly readings based on actual power consumption, in addition to eliminating the need for any personnel to visit and manually take the reading, which not only reduces operational costs but also increases the customers’ privacy. However, in the case of conventional meters, only monthly kWh and kVARh readings are available and no additional information is present, which makes it impossible to detect any anomaly, thereby making system improvement and efficiency enhancement a highly difficult task.

**Fig 13:**
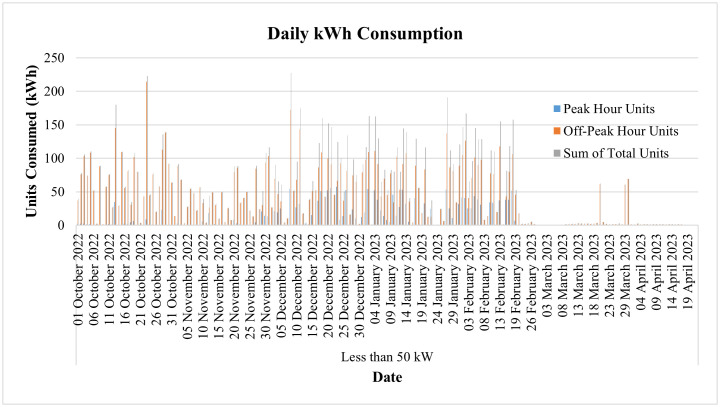
Daily consumption from Sep-22 to April-23 of Load< 50 kW.

The day-to-day consumption data of this connection has been collected through the smart metering database for 8 months, i.e., from September 22 to April 23, and is shown in Fig 13. It represents the consumption of the said connection both during peak and off-peak hours. The timing of peak and off-peak hours for GEPCO consumers is given in Table 2. From the data it is evident that the industry operates during off-peak hours most of the time, and its peak hour consumption is minimal. During the period of 8 months, its maximum one-day energy consumption is noted to be 57.78 kWh in peak hours, while the consumption usually remains less than 10 kWh. On the other hand, the maximum one-day consumption during off-peak hours is noted to be 214.48 kWh during the same period. The graph also shows that during these 8 months, its minimum consumption was observed in the last two months, i.e., March and April. These two months observed the holy months of Ramadan and Eid-ul-Fitr, and the industry remained dysfunctional during this period.

**Table 2:** Timings for peak and off-peak hours.

Month	Peak time	Off-peak time
June to August	7:00 pm to 11:00 pm	Remaining 20 hours
September to November	6:00 pm to 10:00 pm	Remaining 20 hours
December to February	5:00 pm to 9:00 pm	Remaining 20 hours
March to May	6:00 pm to 10:00 pm	Remaining 20 hours

**Fig 14:**
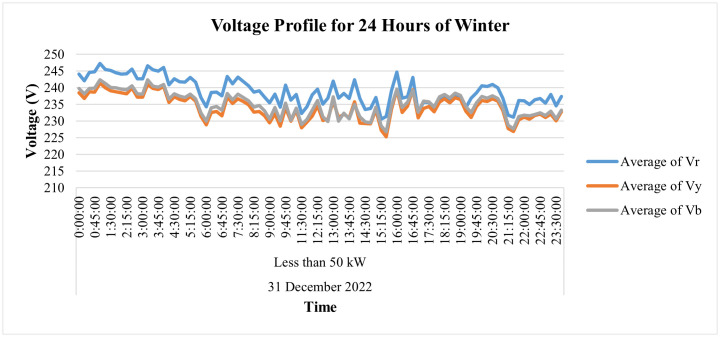
Voltage-profile for one specific day of winter season of load Load< 50 kW.

**Fig 15:**
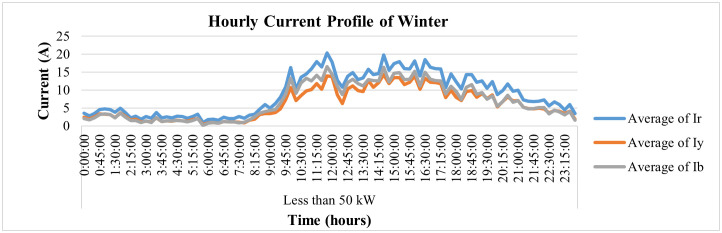
Hourly current-profile of winter season of load< 50 kW.

**Fig 16:**
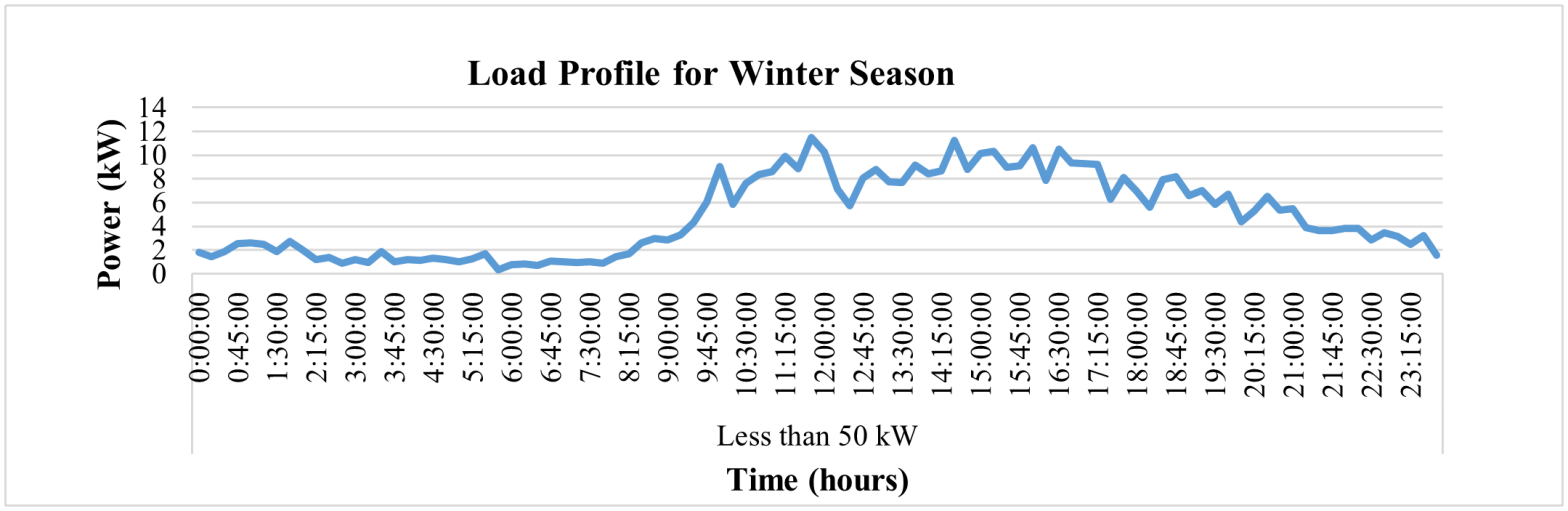
Load-profile of winter season of load< 50 kW.

Moreover, deep analysis of energy parameters can be performed using smart metering data. The analysis of same said industry for winter and summer seasons has been performed in this study at 15-minute interval to deeply analyze the variations in its energy parameters. To analyze winter season, the parameters for Dec 2022 have been observed for this industry. Fig 14 depicts the voltage variations for 24 hours for a specific winter day, i.e., 31 December 2022, where it is evident that the red-phase voltages of this connection show some deviations from their prescribed limit of ±5% of nominal value of 230 V. The blue phase voltages observe only 2 spikes above this prescribed limit while the voltages of yellow phase remain within this prescribed limit of ±5% as required by IEC according to its power quality standard IEC 61000-2-4. On the other hand, Fig 15 and Fig 16 demonstrate hourly current and power consumption of this industry respectively during winter season. The information about current and power consumption helps determine the energy usage behavior of the consumer. For instance, it can be observed that the maximum load of the industry is turned on from 10 am to 6 pm and remains negligible before and after this period.

**Fig 17:**
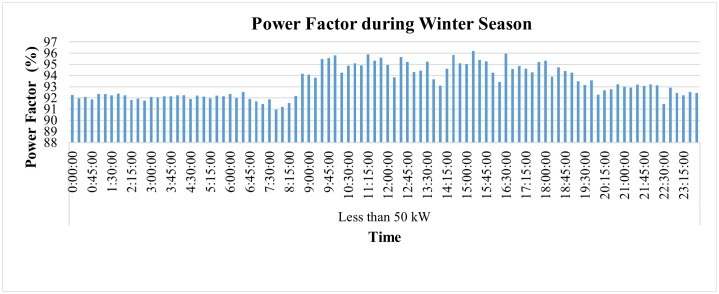
Hourly power factor during winter season of Load< 50 kW.

**Fig 18:**
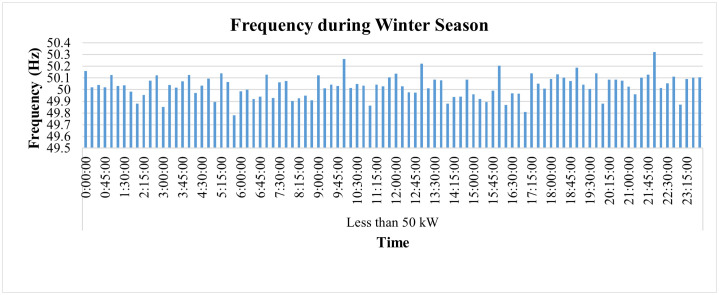
Hourly frequency variations during winter season of Load< 50 kW.

The SMs also enable to monitor and analyze PF and frequency as shown in Fig 17 and Fig 18 respectively, where PF, on average, remains higher than 0.9 which is not only essential to maintain PQ but also leads to impose penalty to the consumer by GEPCO in case his PF get lower than this value. However, its continuous monitoring immediately indicates its variations and provides an opportunity to take timely action to improve it. The frequency of this industry, however, remains within prescribed limit of ±1% of nominal value of 50 Hz as shown in Fig 18.

**Fig 19:**
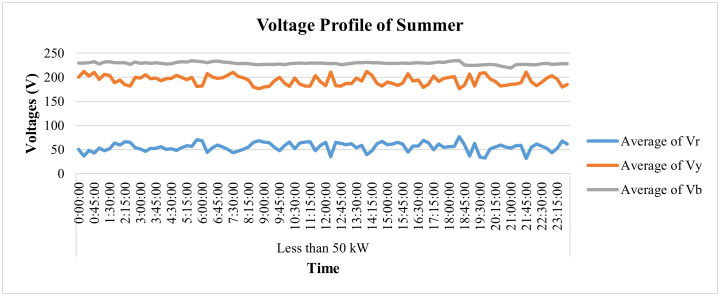
Voltage-profile for one specific day of summer season Load< 50 kW.

In addition to winter season, the PQ parameters of this industry are also analyzed for summer season. For the said purpose, its PQ parameters are observed for May 2022. The 24-hour data of voltages at 15-minute interval for one specific summer day, i.e., 20 May 2022 has been taken through SM database and plotted in Fig 19 for in-depth analysis of voltage variations, where it can be observed that the blue-phase voltages remained stable and within the permissible limit throughout the day. The voltages of the yellow phase, however, were disturbed and remained between 175V and 212V all day. Similarly, red-phase voltages also remained quite below the acceptable range and were observed to be between 31V and 76V throughout the day.

**Fig 20:**
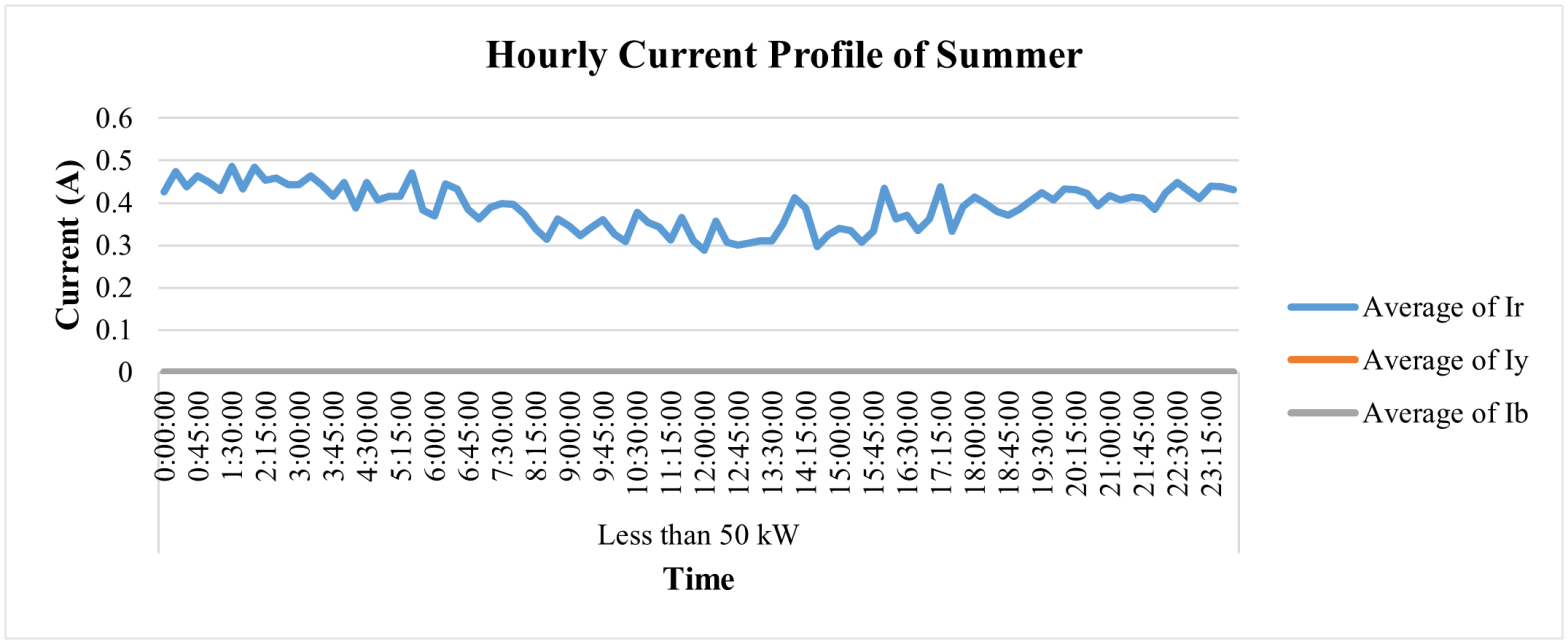
Current profile for one specific day of summer season Load< 50 kW.

**Fig 21:**
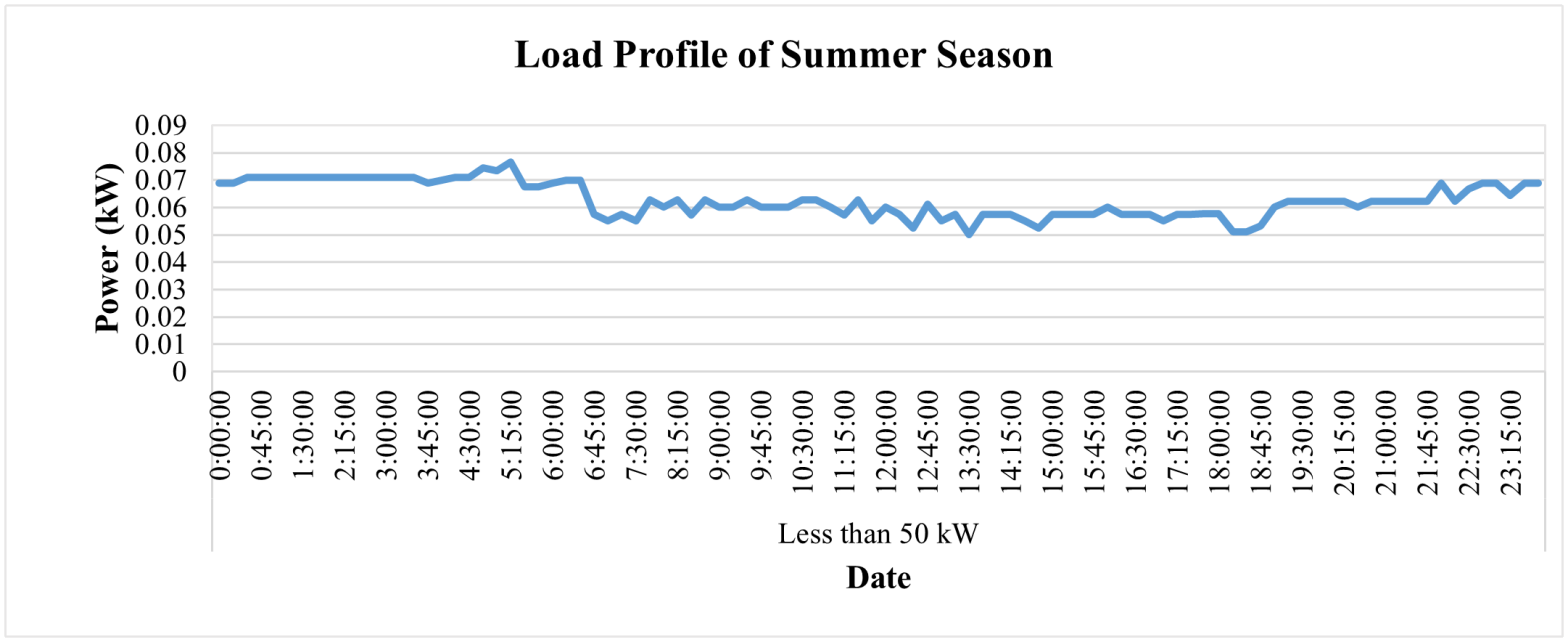
Load-profile for summer season for Load< 50 kW.

Additionally, its average current patterns throughout the day are shown in Fig 20, and its load profile is given in Fig 21. It can be observed that the current of both yellow and blue phases remained zero while minimal current flowed only in the red phase, which depicts that this industry was closed in the month of May and only some single-phase light loads were operated by the consumer. Fig 21 shows power consumption was also minimal in this period, which validates the depiction of Fig 20.

**Fig 22:**
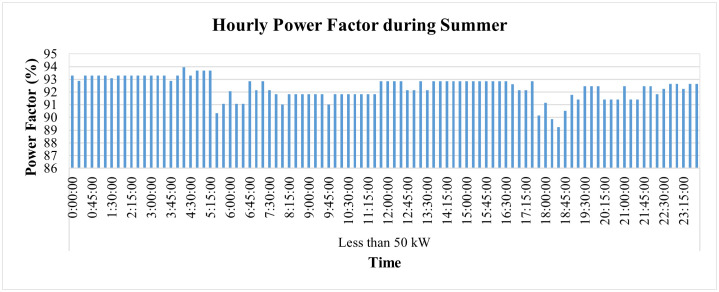
Hourly power-factor during summer season for Load< 50 kW.

**Fig 23:**
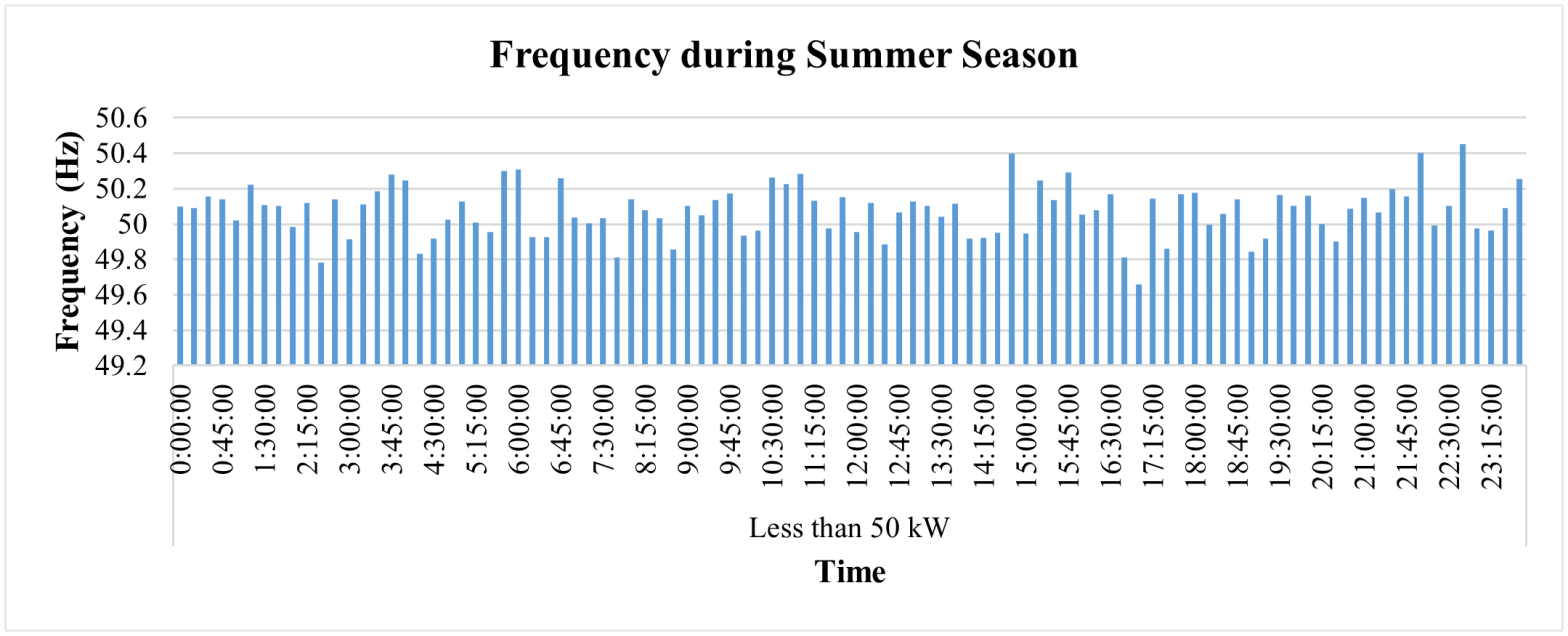
Hourly frequency variations during summer season for Load< 50 kW.

The hourly PF of this connection during the summer season, on average, remains above 90%, as shown in Fig 22. The PF, however, went slightly lower than the target of 90% twice, first at 6:15 p.m. and then at 6:30 p.m., when it reached 89.8% and 89.2%, respectively. On the other hand, its hourly frequency variations remain within the prescribed limit of ±1% of nominal value, as represented in Fig 23.

The continuous monitoring of these PQ parameters is a prerequisite to improve and maintain good PQ. It gives insight into very useful information which is very helpful in energy planning and forecast, resource allocation and distribution, offering time of use (ToU) tariff, grid optimization, and energy management instead of just focusing on enhancing power generation capacity in proportion to increased energy demand. Moreover, information provided by SMs regarding sudden consumption changes along with consumer usage patterns not only alert the utility but can also help it introduce multiple tariff structure for different industries to decrease peak demands in specific times eliminating the need of superfluous generation.

#### 4.4.2. Case study II: Load category between 50 kW and 500 kW..

In this section, we will study and analyze industrial connection with a sanctioned load above 50 kW and below 500 kW. For the said purpose, the connection in the name of Warraich Rice Mills bearing Reference Number 30 12227 0002500 has been selected, which has a sanctioned load of 320 kW and utilizes CTs of ratio 800:5 A.

**Fig 24:**
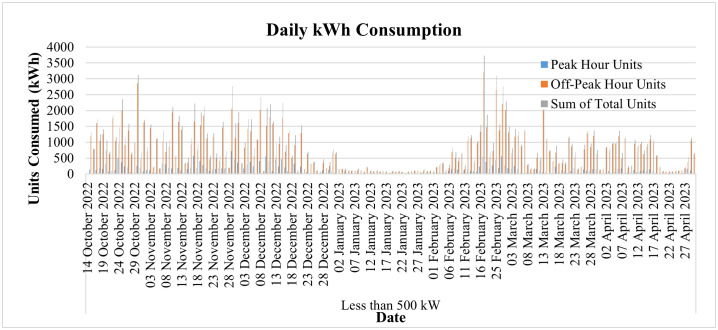
Daily consumption from Oct-22 to April-23 of 50 kW ≤ Load ≤ 500 kW.

The day-to-day power consumption data of this consumer has been collected through the MDMS database for 7 months, i.e., from October 14, 2022, to April 30, 2023, and is depicted in Fig 24. The graph also shows that during these 7 months, its minimum consumption is observed in the month of January, when none of the major industrial loads is operational. The load is again observed to be reduced to its lowest from April 18 to April 28, and upon investigation it is revealed that the load reduction is due to the holy occasion of Eid-ul-Fitr. In the case of conventional meters, as already discussed, there is no concept of day-to-day readings, and the utility must wait for the entire month to see the consumption patterns. Therefore, the utilities are not able to monitor if the industry is showing any anomalies, and thus power theft and system quality remain at risk.

**Fig 25:**
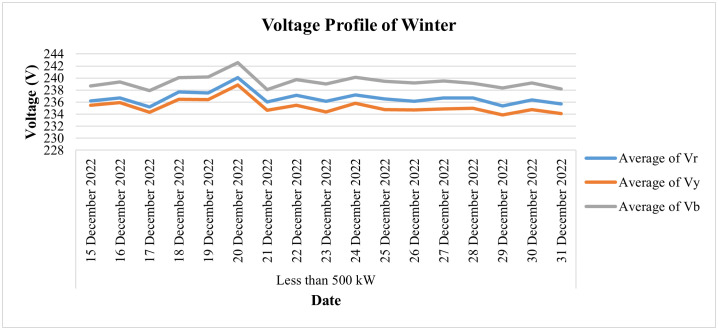
Daily voltage-profile for winter season of 50 kW ≤ Load ≤ 500 kW.

The daily voltage average of Warraich Rice Mills during the last two weeks of the month of December is shown in Fig 25 which shows that during these two weeks, the value of daily average voltages remained within the prescribed limit of ±5% for all three phases except blue-phase voltages, which observed a slight deviation on the 20^th^ December when average voltages of this phase reached 242.5V.

**Fig 26:**
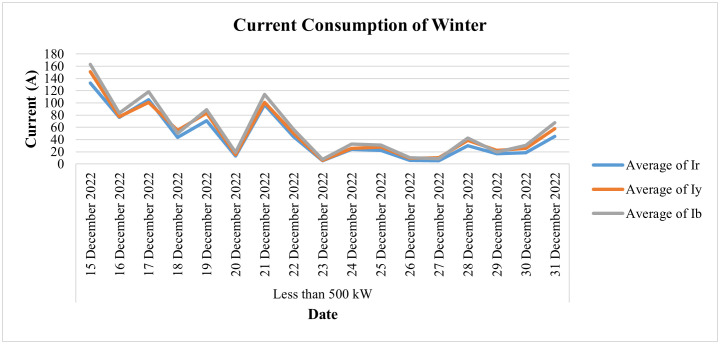
Daily current-consumption for winter season of 50 kW ≤ Load ≤ 500 kW.

**Fig 27:**
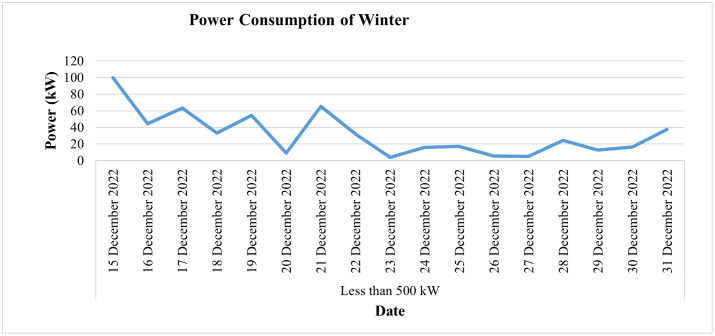
Average power-consumption for winter season of 50 kW ≤ Load ≤ 500 kW.

Fig 26 depicts the daily current consumption of this consumer for the winter season (i.e., for the last 2 weeks of December 2022), whereas Fig 27 shows the power consumption behavior for the same period. From the data, we can observe sharp dips in current and power consumption on December 20 and December 23, along with minimal consumption on December 27. This clearly indicates fluctuations in the load pattern for this category. Notably, December 23 was a Friday, a day when the industry typically remains closed. Additionally, the load shows a significant decline starting from December 21, further highlighting the irregular consumption during this period. It is a rice mill, has a seasonal connection, and operates at its maximum load in the winter from October to December. Its load is reduced during the second half of December and remains minimal in January.

#### 4.4.3. Case study III: Load category > 500.

In this section, we will study and analyze an industrial connection with a sanctioned load above 500 kW. For this, an electricity connection in the name of Mohammad Nawaz S/O Ali Hussain bearing Reference Number 30 12227 0015007 has been selected that utilizes CTs of ratio 100:5 A, PT of ratio 11000:110 V, and has a sanctioned load of 975 kW.

**Fig 28:**
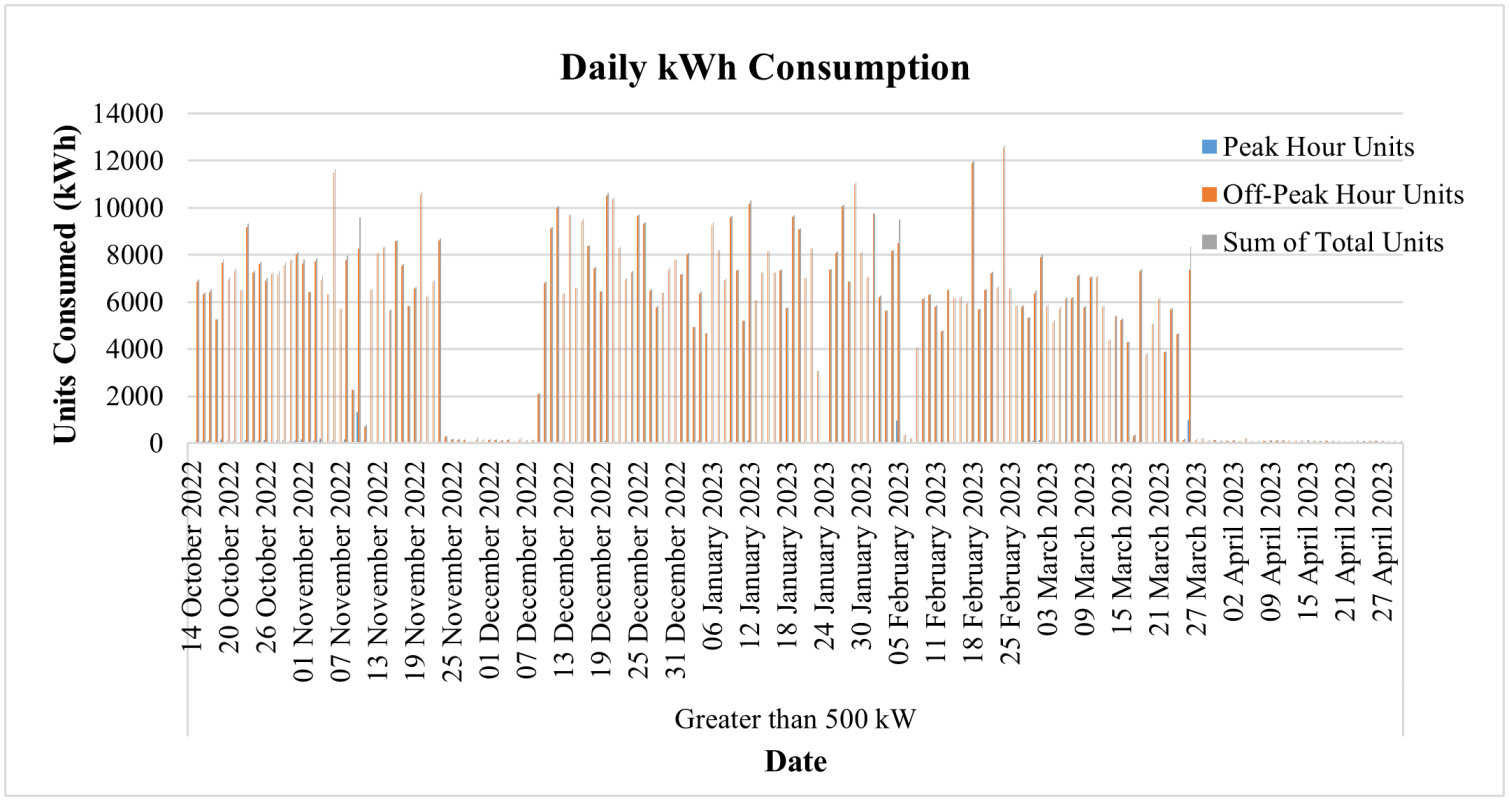
Daily consumption from Oct-22 to April-23 of Load category > 500.

The data on day-to-day power consumption in this industry has been collected through the MDMS database for 7 months, i.e., from October 14, 2022, to April 30, 2023, and is depicted in Fig 28, where it can also be observed that the industry remained closed from November 24 to December 9 and showed minimal consumption only. The industry was also closed for the whole month of April, the month of the holy occasion of Eid-ul-Fitr, and, therefore, shows no energy consumption.

**Fig 29:**
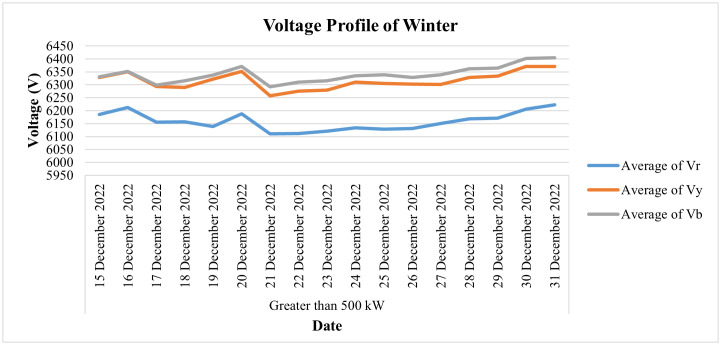
Daily voltage-profile for winter season of Load category > 500.

**Fig 30:**
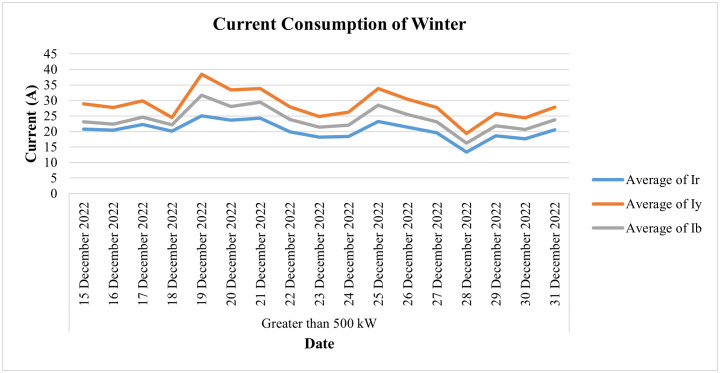
Daily current-consumption for winter season of Load category > 500.

**Fig 31:**
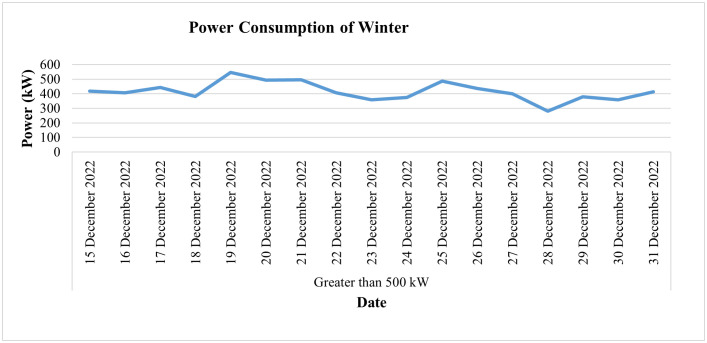
Average power-consumption for winter season of Load category > 500.

This industry is fed by an 11 KV electric supply (or 6.351 KV phase to neutral), which is to be within the prescribed limit of 5% of its nominal value as per the IEC standard. Fig 29 shows the daily voltage average of this connection during the last two weeks of the month of December, which may be helpful in analyzing its voltage profile during the winter season. It shows that during these two weeks the value of daily average voltages remains within the prescribed limit of 5% of their nominal value for all three phases. On the other hand, the daily current consumption of this consumer for the winter season (i.e., for the last 2 weeks of December 2022) and the average power consumption behavior of this consumer for the same period are depicted in Fig 30 and Fig 31, respectively, where slight dips in current and power consumption can be seen on the 16^th^, 23^rd^, and 30^th^ of December because these are Fridays and the industry observes half days each Friday.

**Fig 32:**
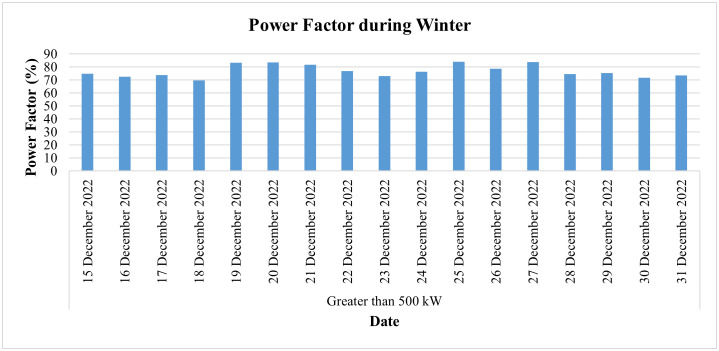
Daily average Power Factor during winter season of Load category > 500.

**Fig 33:**
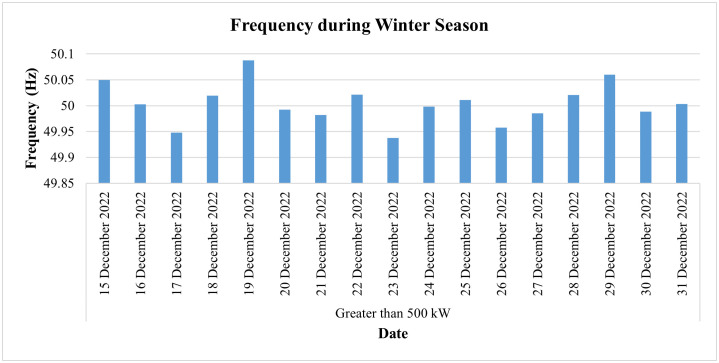
Daily average frequency during winter season of Load category > 500.

Fig 32 represents its PF for the winter season (i.e., for the last two weeks of December) and shows that the PF of this industry remained between 69% and 84%, thus lower than the prescribed limit of 90%. Since the low power factor (LPF) leads to an LPF penalty for the consumer in his electricity bill, this LPF may cause a huge financial loss to the consumer and could be equal to around Rs. 184,275 (one lac eighty-four thousand two hundred and seventy-five rupees) if the monthly PF remains at 69%, while an 84% monthly PF may cause an LPF penalty of around Rs. 52,650 (fifty-two thousand six hundred and fifty). Therefore, continuous monitoring and analyzing PF may lead to protecting the consumer from huge financial losses since it enables facility owners to take necessary and timely action to improve the PF. On the other hand, if we analyze the frequency of this industry as shown in Fig 33, we can see its frequency remained in the range of 49.93 Hz to 50.08 Hz and hence remained within an acceptable range.

**Fig 34:**
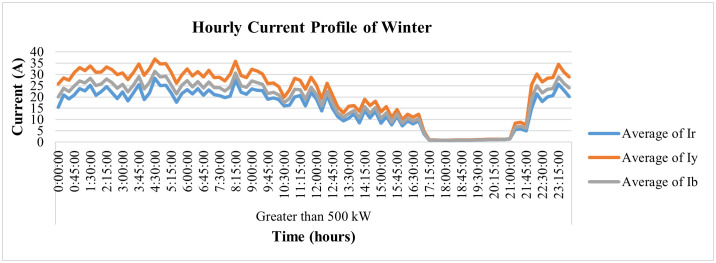
Hourly current-profile of winter season of Load category > 500.

**Fig 35:**
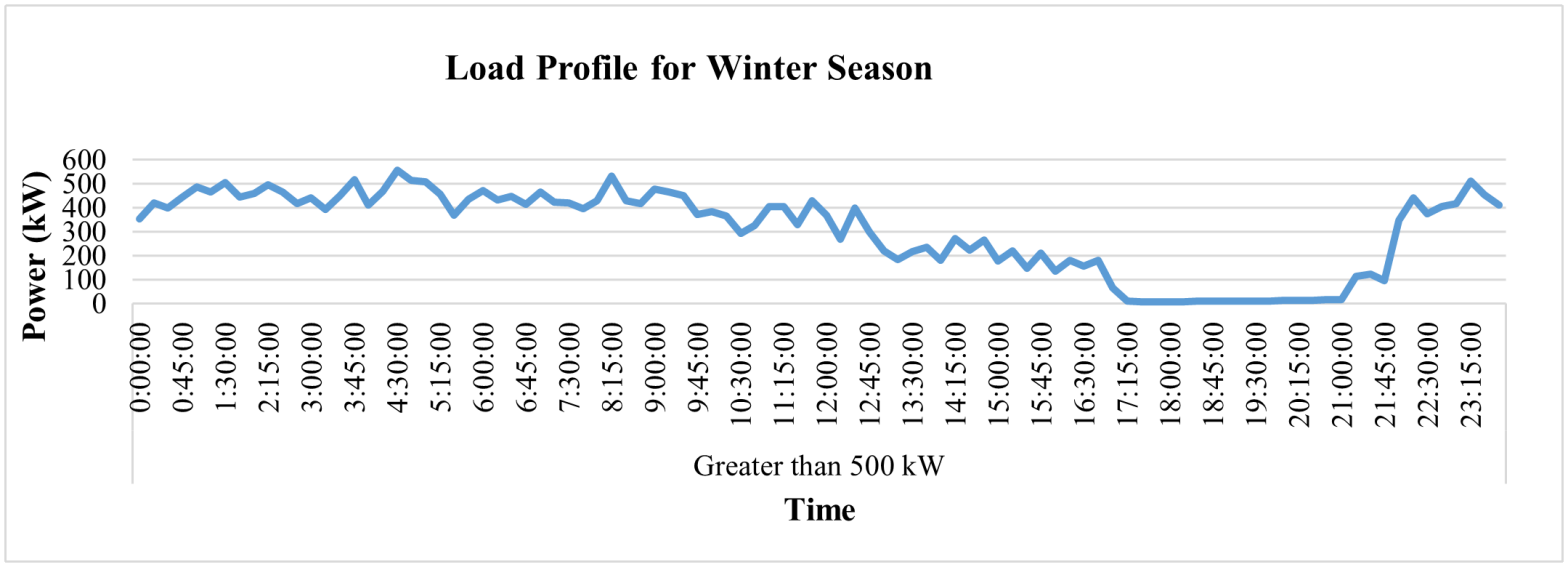
Load-profile of winter season of Load category > 500.

On the other hand, Fig 34 and Fig 35 represent the average hourly current patterns and load profiles for the winter season, respectively. The figures show this industry runs throughout the day, even at night. The industrial processes, however, stopped from 5 pm to 9 pm, i.e., during peak hours, and the load again increased once the peak hours were over.

**4.4.3.1. Power factor improvement: Sultan steel industry case**: As demonstrated earlier, SMs ensure continuous monitoring of PF, just like other energy parameters, and thereby help in its improvement. It has two-fold benefits: one for the consumer, who can gain financial benefits by protecting himself from LPF penalties, and the other for the utility, which can not only avoid unnecessary current flow increases and enhance power losses but also get better energy efficiency.

**Fig 36:**
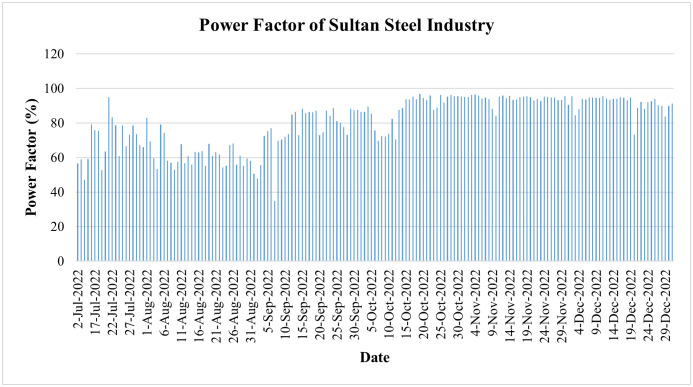
Power factor trend from July 2022 to December 2022 of Sultan steel industry of load category > 500.

This subsection extends Case Study 3 to quantify PF improvement at Sultan Steel (M/S Sultan Steel Industry bearing Reference No. 30122270003300), sanctioned load: 4,950 kW. A SM replaced the conventional meter on 22 June 2022, enabling continuous PQ monitoring. GEPCO’s SM data indicated low average PF of 0.69 in July 2022 and 0.61 in August 2022. Following notification and user training in mid-August, the consumer installed capacitor banks within two weeks. Subsequent records as shown in Fig 36 display sustained PF improvement.

For industrial consumers, when PF<0.90, the penalty equals a 2% increase in fixed charges for each 1% shortfall below 0.90 [41,42]. The monthly PF penalty (Rs) is calculated by Eq 1:


Penalty(Rs)=0.02(90−100PF)F                     (1)

where,

PF= measured average power factor (0–1);

F= monthly fixed-charges base (Rs) defined by the industrial tariff schedule;

Δ=90−100PF= PF shortfall below 0.90 in percentage points (pp).

For the Sultan Steel case, the tariff’s fixed charges are calculated by Eq 2.


F=(sanctioned load)×rate                                     (2)

As sanctioned load is 4,950, so by using Eq. 2 the fixed charges will be Rs 2,227,500.

4,950 kW×Rs 450/kW=Rs 2,227,500.The LPF penalty for PF = 0.69 and PF = 0.61 is calculated using Eq. 1.

At PF = 0.69: Δ=90−69=21pp → penalty =0.02×21×F=0.42F=Rs 935,550.

At PF = 0.61: Δ=90−61=29pp → penalty =0.02×29×F=0.58F=Rs 1,291,950.

Daily average PF was computed from VEE-validated 15-minute intervals and grouped into baseline (≤ 14 Oct 2022) and post-deployment (≥ 15 Oct 2022) periods. Because the two groups have unequal variances and sample sizes, we used Welch’s t-test [43] provided in Eq 3.


$$t\;\;=\;\;\frac{{\bar{\;x}}_{\text{post}}-{\bar{\;x}}_{\text{pre}}}{\sqrt{\frac{s_{\text{post}}^{\text{2}}}{n_{\text{post}}}+\frac{s_{\text{pre}}^{\text{2}}}{n_{\text{pre}}}}}\;$$ (3)

Where;

${\bar{\;x}}_{\text{post}}$— Sample mean of the post-deployment daily PF values (expressed in %).

${\bar{\;x}}_{\text{pre}}$— Sample mean of the baseline daily PF values (in %).

$s_{\text{post}}^{\text{2}}$— Sample variance of post-deployment daily PF (%²).

$s_{\text{pre}}^{\text{2}}$— Sample variance of baseline daily PF (%²).

$n_{\text{post}}$— Sample size in the post period (number of days with valid daily PF).

$n_{\text{pre}}$— Sample size in the pre period (number of days with valid daily PF).

To quantify the practical magnitude of improvement, we report Cohen’s d [44] as provided in Eq 4. Given the strong variance reduction post-deployment, we present the effect using the baseline (pre) SD as a stable reference:


$$\begin{array}{c} d\;\;=\;\;\frac{{\bar{\;x}}_{\text{post}}-{\bar{\;x}}_{\text{pre}}}{s_{\text{pre}}}\; \end{array}$$ (4)

Using the daily PF series described above:

• Substituting the data (baseline n=90, ${\bar{\;x}}_{\text{pre}}=\text{6}\text{7}.\text{5}\%$, $s_{\text{pre}}=\text{1}\text{1}.\text{2}\%$; post-deployment n=78, ${\bar{\;x}}_{\text{post}}=\text{9}\text{3}.\text{6}\%$, $s_{\text{post}}=\text{2}.\text{4}\%$) into Eq. 3 gives


t=16.96

To calculate the effect size, substituting into Eq. 4 gives,


d=2.40

Following the deployment and corrective action, the post-deployment period shows a substantial, statistically significant increase in PF and a marked reduction in day-to-day variability (greater operational consistency) as illustrated in Fig 36. Table 3 provides the daily PF summary statistics before and after the deployment of the capacitor banks in Sultan Steel. These outcomes are consistent with reduced exposure to PF penalties for the consumer and a lower reactive burden and hence potential efficiency gains for the utility.

**Table 3:** Daily power-factor (PF) summary statistics before and after deployment of capacitor banks of Sultan steel.

Period	n	Mean PF (%)	SD (%)	Median (%)	Min (%)	Max (%)
**Pre (≤ 2022-10-14)**	90	67.53	11.18	67.78	47.07	94.99
**Post (≥ 2022-10-15)**	78	93.55	2.44	94.02	84.1	96.93

It is worth observing that LPF results in decreased energy efficiency and enhanced energy losses, as more current is required to produce the specified power. This additional current flow will raise the electrical network’s resistance losses. In addition, LPF necessitates that the utility compensates for inefficient energy usage by providing additional reactive power. The poor PF necessitates an increase in generation in addition to an increase in energy waste. In contrast, a high PF reduces losses, improves energy efficiency, optimizes energy consumption, and reduces the requirement for superfluous expansion of generation, thus leading to a sustainable electricity sector. The adoption of SMs has therefore contributed to the achievement of sustainability by enhancing and improving the PF, as discussed above in this subsection.

Moreover, from the case studies discussed above, it is evident that PQ analytics determine any variation in any of the PQ parameters like voltage dips, frequency, PF deviation, etc., thereby enabling the utility to take proactive or corrective measures to ensure grid stability and maintain the quality of delivered electricity. It also leads to enhanced efficiency of electrical equipment, increases their lifespan, and minimizes energy waste. Furthermore, PQ analytics also determines and addresses the issue of unbalanced loading and unreliable and inefficient distribution of energy, and addressing these factors result in reduced energy losses, decreased energy waste, and increased sustainability in the electricity sector.

### 4.5 Utility-wide power-factor improvement: Normalized scaling and worked example

To translate the observed site-level PF improvement to a utility-level estimate for GEPCO’s industrial segment, we adopt a normalize-then-scale framework [45,46]. First, the case-site improvement is converted to a normalized gain per unit of contracted apparent power; second, a load-weighted average improvement is computed over the industrial segment using conservative coverage/adoption assumptions. This follows standard scalability/replicability logic used in utility case-study extrapolations. It has the following steps.

Step 1 — Normalize the site improvement

The PF gain per MVA is defined in Eq 5 as shown below:


$$\Delta {\text{PF}}_{\text{norm},\text{MVA}}\;\;=\;\;\frac{\Delta {\text{PF}}_{\text{case}}}{S_{\text{site}}}\;$$ (5)

where;

$\Delta {\text{PF}}_{\text{case}}$ = site-level PF uplift (unitless, e.g., 26.1 pp = 0.261)

$S_{\text{site}}$ = site contracted power (MVA)

For the Sultan Steel site, the observed uplift was $\Delta {\text{PF}}_{\text{case}}=\text{0}.\text{2}\text{6}\text{1}\;$(26.1 percentage points) and the contracted apparent power was $S_{\text{site}}=\text{4}.\text{9}\text{5}$; therefore the normalized improvement per unit load is Using Eq 5$\;\Delta {\text{PF}}_{\text{norm},\text{MVA}}=\frac{\Delta {\text{PF}}_{\text{case}}}{S_{\text{site}}}=\frac{\text{0}.\text{2}\text{6}\text{1}}{\text{4}.\text{9}\text{5}}=\text{0}.\text{0}\text{5}\text{2}\text{7}\;\text{PF}\;/\text{MVA}$.

Step 2 — Compute an upper-bound industrial-segment average

If a fraction fof the industrial load is in a “similar large-industrial class” and can realize an improvement approximately equal to the case-site uplift $\Delta {\text{PF}}_{\text{case}}$, while the remainder sees no change, the load-weighted average PF uplift across the industrial segment is defined in Eq 6:


$$\Delta {\text{PF}}_{\text{avg},\text{upper}}\;\;=\;\;f\;\Delta {\text{PF}}_{\text{case}}\;$$ (6)

where;

f∈[0,1] = similar-class load fraction expected to realize an improvement comparable to the case site (coverage)

with f=0.092 (≈ 9.2%),

$\Delta {\text{PF}}_{\text{avg},\text{upper}}=\text{0}.\text{0}\text{9}\text{2}\times \text{0}.\text{2}\text{6}\text{1}\approx \text{0}.\text{0}\text{2}\text{4}$ (2.4 pp).

Step 3 — Apply a realistic adoption coefficient

Recognizing that not all eligible customers will achieve full improvement immediately, apply an adoption coefficient a to reflect partial roll-out and heterogeneity as provided in Eq 7:


$$\Delta {\text{PF}}_{\text{avg},\text{realistic}}\;\;=\;\;a\;\Delta {\text{PF}}_{\text{avg},\text{upper}}\;$$ (7)

where;

a∈[0,1] = adoption coefficient reflecting partial roll-out and heterogeneous performance (realization)

$\Delta {\text{PF}}_{\text{avg},\text{upper}}$ = industrial-segment upper-bound average PF uplift

$\Delta {\text{PF}}_{\text{avg},\text{realistic}}$ = realistic average PF uplift (after adoption)

with a=0.60,

$\Delta {\text{PF}}_{\text{avg},\text{realistic}}=\text{0}.\text{6}\text{0}\times \text{0}.\text{0}\text{2}\text{4}\approx \text{0}.\text{0}\text{1}\text{4}\text{4}$(1.4 pp).

The normalized gain at the case site is 0.0527 PF/MVA, and under conservative coverage (f=0.092) and adoption (a=0.60) assumptions, the industrial-segment average PF uplift is approximately 1.4 percentage points, providing a transparent, conservative scaling of the measured site-level improvement.

## 5. Advantages of smart metering in the power sector of Pakistan

Based on the GEPCO deployment presented in the manuscript which includes three categories of industrial sanctioned-load, IoT/AMI architecture in four tiers, and MDMS, the following benefits are either directly seen in our case studies, or are operational applications facilitated by the same data and workflows [47–49]:

- Accurate Load Profiling

SMs gather RT information on power consumption. The availability of these load profiles provides useful information in load forecasting, the load planning, infrastructure investment, energy-efficiency programs and grid optimisation. These benefits eventually lead to a more stable, strong, and energy-efficient and sustainable electricity infrastructure in Pakistan.

- Grid Optimization

SMs help DISCOS to receive RT, granular information on energy consumed, voltage, current, frequency, PF, and load profiles, therefore, informing grid optimization, load forecasting, and infrastructure upgrades. Such optimization is critical in minimizing losses, reducing the requirement to augment the generation capacity of power, maximizing the energy efficiency, and, as a result, advancing on a long-term electricity sustainability in Pakistan.

- Reducing the Energy Crisis.

Energy shortage has been a long-term challenge faced by Pakistan and is characterized by power outages and unstable grid. This crisis is increased by population growth and rapid urbanization which bring about more demands on the already scarce resources. The RT data accumulation of SMs enables DISCOS to optimize the production process of electricity and enhance the forecasting of the demand. DISCOS can encourage off-peak demand using ToU pricing by determining peak demand times, which will improve grid stability and sustainability.

- Improving Grid Reliability

Power supply in Pakistan is still prone to failure, power cuts, and instability. With the surveillance of SMs, utilities are able to predict and react timely to possible disturbances and thereby maximize grid stability and reliability. This potential is important to the economic operations of the country which have been plagued in the past by a shaky power supply.

- Environmental Benefits

Pakistan is always in the top ten most climate-prone countries in the world based on the Climate Risk Index (CRI) where it has recorded more than 10,000 deaths as a result of climate-related disasters and around US 4 billion in climate-related disaster damage over the last twenty years. This weakness highlights the need to switch to a more sustainable energy model in terms of environmental sustainability. SMs may contribute greatly by minimizing energy wastage, maximizing energy efficiency, and optimizing grids to decrease the further generation, greenhouse-gas emissions, and conserve the environment which will reduce the mitigation of climate change and the sustainable electricity sector [50].

**Fig 37:**
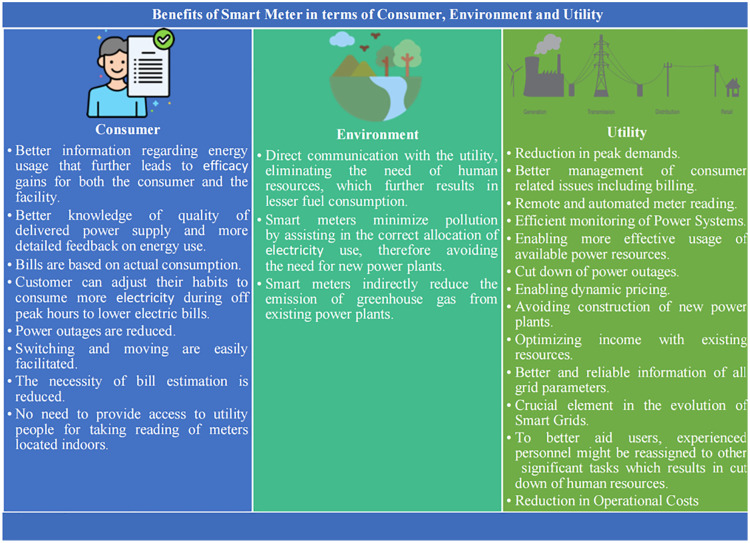
Benefits of SMs from the prospective of consumer, environment and utility/DISCOS.

Fig 37 displays the advantage of SMs in terms of the consumer, environment and utility/DISCOS.

## 6. Challenges and further directions in the implementation of SMs in Pakistan: A contextual perspective

Although smart metering is a revolutionary opportunity to Pakistan power industry, it does not come without challenges. These are challenges that are a combination of technical, institutional, financial, and societal factors peculiar to the energy situation of Pakistan.

1. Public and Utility Acceptance: In general, there is still a lack of awareness to the consumers and even within the utility companies regarding the potential advantage of smart metering, which includes RT monitoring, energy saving, and accurate billing. Several consumers relate SMs with high tariffs, that’s why they resisted. The utilities with their manual processes and old systems tend to be characterized by institutional inertia and being unwilling to change to automated systems depending on the new workflows and performance responsibility.
2. Regulatory and Institutional Gaps: The regulatory framework of Pakistan is still being changed to accommodate AMI. Regulatory push or incentive to make DISCOs embrace quality-oriented and customer-oriented service models is minimal. The lack of, performance-based incentives and policy requirements of SG integration retards adoption. Moreover, there is a lack of development of consumer protection and data privacy laws and inconsistent interoperability technical standards.
3. Lack of Qualified Technical Labor Force: A shortage of technical capacity is observed at most of the utility firms, particularly in underdeveloped and developing countries. To introduce and operate supporting IoT-based smart metering systems, engineers, data analysts, and IT specialists are required, which is scarce in the pool of talents around the public utility. This is yet to be met with training and capacity-building programs.
4. High Upfront Investment: The SMs and the accompanying infrastructure (communication networks, data centers, MDMS, etc.) require considerable capital investment. To cash strapped utilities that are under a circular debt burden, such spending is perceived to be risky. Large-scale deployment requires a significant amount of time to procurement, testing, and phased rollouts, which are delayed by financial constraints.
5. Higher SM Unit Costs and Domestic Production Hurdles: SMs, especially those with advanced analytics, are significantly costlier than traditional electromechanical meters. Dependence on imports, volatile exchange rates and lack of local manufacturing capability make it a costly burden and mass scale deployment is expensive without subsidy & foreign funding assistance.
6. Data Management and Cybersecurity Concerns: The SMs produce tera-byte daily RT data, making robust IT infrastructure needed for its secure storage, processing and analytics. The utilities of Pakistan currently do not have modern data centers and data governance policies. Concerns around data misuse, cybersecurity threats and privacy breaches are on the rise but are not yet adequately matched by institutional safeguards and awareness.

Addressing these challenges requires a coordinated and multi-stakeholder effort by the government bodies (e.g., NEPRA, MoW&P), DISCOs, academia and the private sector. Research on awareness campaigns, capacity building, local technology development, and regulatory reform will be necessary in the process of unlocking the full potential of smart metering in the energy transition of Pakistan.

### 6.1 Future directions for successful smart meter deployment

To implement the SM technology successfully across Pakistan’s power sector, a number of critical aspects should be taken into consideration:

1. Public Awareness and Engagement: The country should have a national campaign to inform the people of the advantages of SMs. It can really help dispel myths like the idea that they hike up tariffs or invade privacy. Utilities, media, and schools can work together to spread the word and get everyone on board. Consumer participation and acceptance is the key to the success of this initiative.
2. Investment and Support: The government and utility companies should ensure that there is adequate investment in the infrastructure necessary for smart metering technology. International cooperation and funding can also be explored for this purpose.
3. Regulatory Framework: A strong regulatory framework is needed for SMs, including data protection. For example, end-to-end encryption can be set for SM data transmissions, strict authentication mechanisms and access controls can be put in place, security audits can be done regularly, certain time limits can be specified for which SM data may be kept, and consumer groups and other relevant stakeholders can be involved in the development or review of data privacy and security policies.
4. Capacity Building and Technical Training: Human capacity remains one of the most critical enablers of smart metering success. Comprehensive training programs are needed for engineers, IT professionals, and field technicians responsible for installation, maintenance, and data analytics. Universities and technical institutes can play a vital role in designing courses that bridge the gap between traditional electrical engineering and emerging digital technologies such as IoT, cloud analytics, and cybersecurity. A well-trained workforce will ensure continuity of operations, effective troubleshooting, and sustainable system performance.

The long-term success of smart metering in Pakistan will depend on aligning technology deployment with social awareness, regulatory oversight, and institutional capacity. By combining technical innovation with transparent governance and skilled human resources, Pakistan can position smart metering as a cornerstone of its energy modernization agenda.

### 6.2 Future analytical extensions (Machine learning and deep learning)

This paper focuses on the design, field implementation, and operational validation of the IoT-based AMI system, the resulting dataset may provide a strong foundation for advanced quantitative analyses.

We plan to explore, as appropriate, machine-learning approaches for loss forecasting and anomaly detection, and deep-learning models for short-term load forecasting, outage prediction, and voltage-profile reconstruction. For DSM, we will investigate clustering and reinforcement-learning strategies for segmentation, demand-response targeting, and peak-load shifting. We also intend to assess power-quality classification using supervised models to detect sags, swells, harmonics, and frequency anomalies. We will also study economic/tariff analytics by linking AMI time series with PF-related penalties and incentives to estimate utility-level impacts.

## 7. Conclusion

Smart metering is a crucial step in addressing the ongoing operational and structural challenges facing by the electricity sector of Pakistan. Adoption of smart metering technology may support energy conservation, reduce non-technical and technical losses, enhance billing transparency, and improve customer satisfaction. The RT data generated by SMs capacitate both DISCOS and customers to make learned and data-driven decisions on the consumption and management of energy. The case studies presented in this research demonstrate how RT, granular data of SMs contributes to billing statistics, load profile analytics, PQ analytics, and consumer behaviour analytics. These data streams facilitate utilities’ ability to identify voltage fluctuations, pinpoint LPF anomalies, and enact prompt remedial actions. Further, it shows how the deviated PQ parameters are timely identified using SMs, and their timely rectification leads to potential cost savings. For instance, daily PF improved significantly in one large industrial case from 67.5% to 93.6% percent that reflects measurable operational gains and reduced LPF related penalties. The observed improvement, validated through Welch’s t-test with a large effect size, also indicates reduced reactive loading on the DISCOS network. Such outcomes demonstrate that data-informed interventions can yield tangible efficiency and reliability benefits. Thus, the deployment of SMs in Pakistan will contribute to enhancing energy sustainability by reducing losses and improving efficiency. Moreover, it helps preserve the environment by reducing the carbon emissions, improving air quality, and developing cleaner energy practices. The implementation of smart metering technology is vital to upgrading and transforming Pakistan’s electricity sector and harmonizing it with global sustainability plans. Using the normalize-then-scale technique, a conservative utility-wide estimate of PF uplift is obtained of approximately 1.4 percentage points on the reported coverage/adoption terms, on the industrial-segment. In future, we aim to extend dataset to explore advanced analytical approaches such as predictive modeling, cost–benefit assessment, and scalability analysis in collaboration with DISCO and policymakers. While the present work focuses on design, deployment, and performance evaluation, it establishes a foundation for subsequent research aimed at achieving, data-driven grid optimization in Pakistan’s evolving power landscape.

## Supporting information

S1 File Weekly aggregated smart-meter operational dataset. (XLSX)

S2 File Weekly aggregated peak and off-peak energy consumption dataset. (XLSX)
